# IL-12 stimulates CTLs to secrete exosomes capable of activating bystander CD8^+^ T cells

**DOI:** 10.1038/s41598-017-14000-z

**Published:** 2017-10-17

**Authors:** Lei Li, Steven M. Jay, Yan Wang, Shu-Wei Wu, Zhengguo Xiao

**Affiliations:** 1 Department of Animal and Avian Sciences, University of Maryland, College Park, Maryland 20742 USA; 2 Fischell Department of Bioengineering, University of Maryland, College Park, Maryland 20742 USA; 3 Department of Cell Biology and Molecular Genetics, University of Maryland, College Park, Maryland 20742 USA

## Abstract

An effective cytotoxic T lymphocyte (CTL) response against intracellular pathogens is generally accomplished by immense CTL expansion and activation, which can destroy infected cells. Vigorous immune responses can lead to activation of bystander CD8^+^ T cells, but the contribution from antigen-specific CTLs is not well understood. We found that CTLs secrete extracellular vesicles following antigen stimulation. These CTL-derived vesicles contain CTL proteins and exhibit markers and size profiles consistent with exosomes. Interestingly, further stimulation of CTLs with IL-12 impacts exosome size and leads to selective enrichment of certain exosomal proteins. More important, exosomes from IL-12-stimulated CTLs directly activated bystander naïve CD8^+^ T cells to produce interferon-γ (IFNγ) and granzyme B (GZB) in the absence of antigens, whereas control exosomes derived from antigen-stimulated CTLs did not. In addition, IL-12 induced exosomes are able to strengthen the effects of weak antigen stimulation on CTLs. Proteomic analysis demonstrates that IL-12 stimulation alters catalytic and binding activities of proteins in CTL exosomes. Our findings indicate that the biological function and morphology of exosomes secreted by CTLs can be influenced by the type of stimulation CTLs receive. Thus, a fully functional, ongoing, antigen-specific CTL response may influence bystander CD8^+^ T cells through secretion of exosomes.

## Introduction

One of the body’s primary responses to infection is the activation of cytotoxic T lymphocytes (CTLs), which undergo drastic expansion and become effectors that destroy pathogen-infected cells. Communication between antigen-specific and non-specific immune cells is critical to the ability of the immune system to mount a vigorous adaptive immune response while maintaining functional innate and adaptive immunity against other pathogens. While much important knowledge has been uncovered^1,2^, understanding of CTL intercellular communication mechanisms remains incomplete. Advancing this understanding may lead to improvement in the design of immunotherapies in a variety of applications, such as chronic infections and cancers.

One validated mechanism of CTL intercellular communication is via extracellular vesicles, particularly exosomes^3^. Exosomes are membrane-bound vesicles secreted by somatic cells^4–8^, including T cells and B cells, that range in size from 30 to 150 nm^3^. Exosome formation can be driven by two pathways; exosomal sorting complex required for transport (ESCRT)-dependent^9,10^, and ESCRT-independent^3,11,12^. Exosome secretion may be constitutive, as in most cancer cells, or regulated, as in T and B cells, which require receptor stimulation^3,13–15^. Exosomes are effective immune regulators based on their unique features: small size enabling rapid and unadulterated horizontal transfer of materials between cells; enclosed environment to protect cargo (proteins and RNAs) from degradation during transport; and ability to fuse with biological membranes.

Production of exosomes by T cells only occurs following T cell activation^13–16^. The biological function of exosomes is thought to be related to the proteins^3^ and/or RNAs^17^ contained therein. Exosomes from CD8^+^ T cells have been shown to inhibit HIV transcription *in vitro* ^18^. In addition, human T cells secrete exosomes following activation *in vitro* that enhances IL-2-mediated immune responses in naïve CD8^+^ T cells, suggesting that activated T cells (both CD4^+^ and CD8^+^) may specifically communicate with resting, bystander T cells via exosomes^19^. In mice, antigen-stimulated CD8^+^ T cells secrete exosomes that enhance the metastasis of melanoma cells to the lung via Fas signaling triggered by the exosome protein FasL^20^. However, it remains unknown if and how variations in CTL-derived exosome functions are associated with differences in CTL stimulation.

Full activation of CTLs requires three discrete signals: antigen (1), costimulation (2), and inflammatory cytokines (3), such as IL-12^21^. Here, we investigate the hypothesis that exosomes secreted by activated CTLs differ based upon the stimulation from signal 3. Specifically, we focused on CTL stimulation by the cytokine IL-12, which has been shown to be an important third signal cytokine in murine models^21,22^. To test this hypothesis, we used OT-I transgenic CD8^+^ T cells in an *in vitro* system. Our results demonstrate that IL-12 induces structurally and functionally distinct activated CTL-derived exosomes, which can thereby activate bystander CD8^+^ T cells without the presence of antigen.

## Results

### IL-12 stimulation impacts activated CTL-derived vesicle size

**Figure 1:**
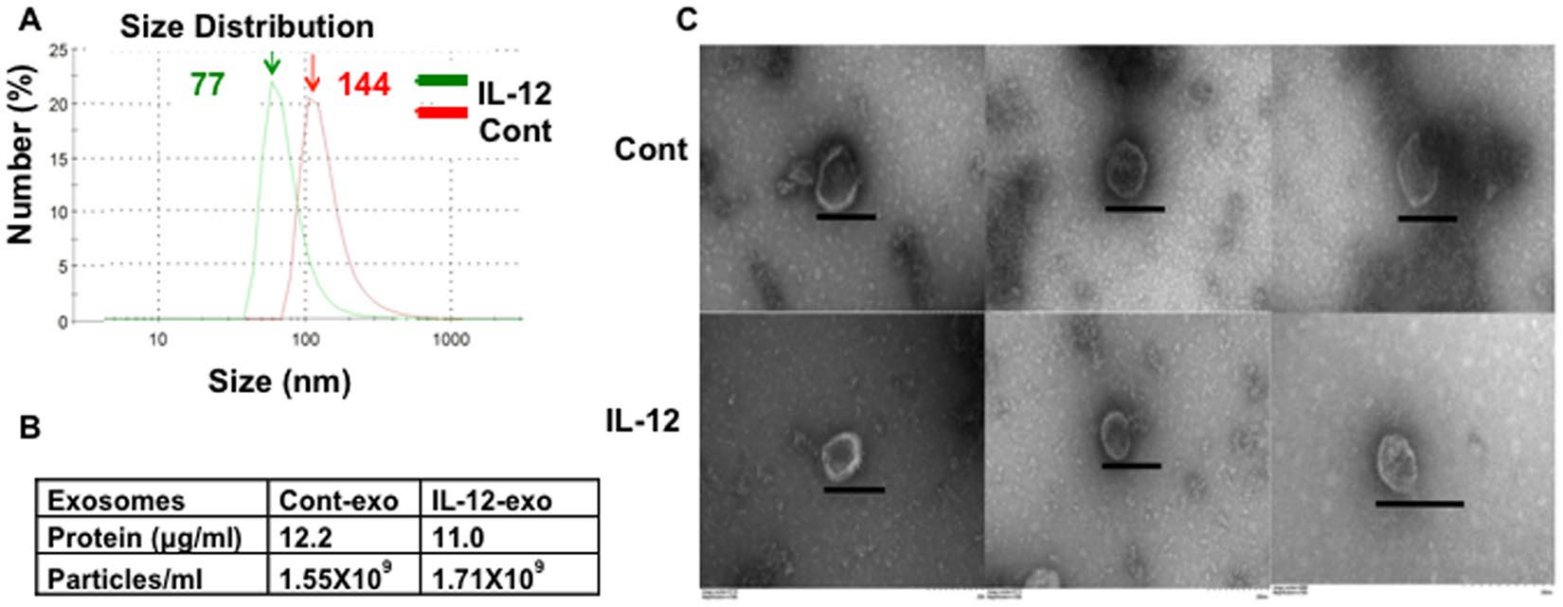
Characterization of CTL-derived vesicles. Naïve CD8^+^ T cells purified from OT-I mice were stimulated with 2SI (cont) or 3SI (IL-12) for three days *in vitro*, and extracellular vesicles were purified from each supernatant. **(A**) Size distribution measured in Malvern Zetasizer Nano ZS90. (**B**) Concentration of protein and vesicle particles per mL of supernatant. (**C**) Transmission Electron Microscopy. Purified vesicles were observed under Zeiss EM10 transmission electron microscope. The graphs vary on amplification magnitude, and the bar in each graph indicates 200 nm. Each experiment was replicated at least three times with similar results.

Purified naïve CD8^+^ T cells from OT-I mice were stimulated with antigen and costimulation (2 signals-2SI) or 2SI plus IL-12 (3 signals-3SI) in vesicle-depleted media^23,24^. Extracellular vesicles were purified from supernatant three days following stimulation^25–27^ and demonstrated size ranges (Fig. 1A) and morphology (Fig. 1C) consistent with exosomes. The vesicles derived from 2SI-activated CTLs (cont-exo) were larger than 3SI-conditioned CTL-derived vesicles (IL-12-exo), with mean sizes of 144 and 77 nm, respectively (Fig. 1A). In both populations, protein content was elevated at 12.2 and 11.0 μg/mL (Fig. 1B) as compared to supernatant from non-activated cells (~0.1 μg/mL)^25,28–30^. Vesicle concentrations from the two activated cell populations were also similar, at 1.55 and 1.71 × 10^9^/mL in supernatant, respectively, as detected by a NanoSight LM10^31^ (Fig. 1B), consistent with similar data from other cell types^25,28,31,32^. Therefore, the morphology of extracellular vesicles from antigen-stimulated CTLs appears to resemble that of exosomes.

### Activated CTL-derived vesicles express typical exosome markers

**Figure 2:**
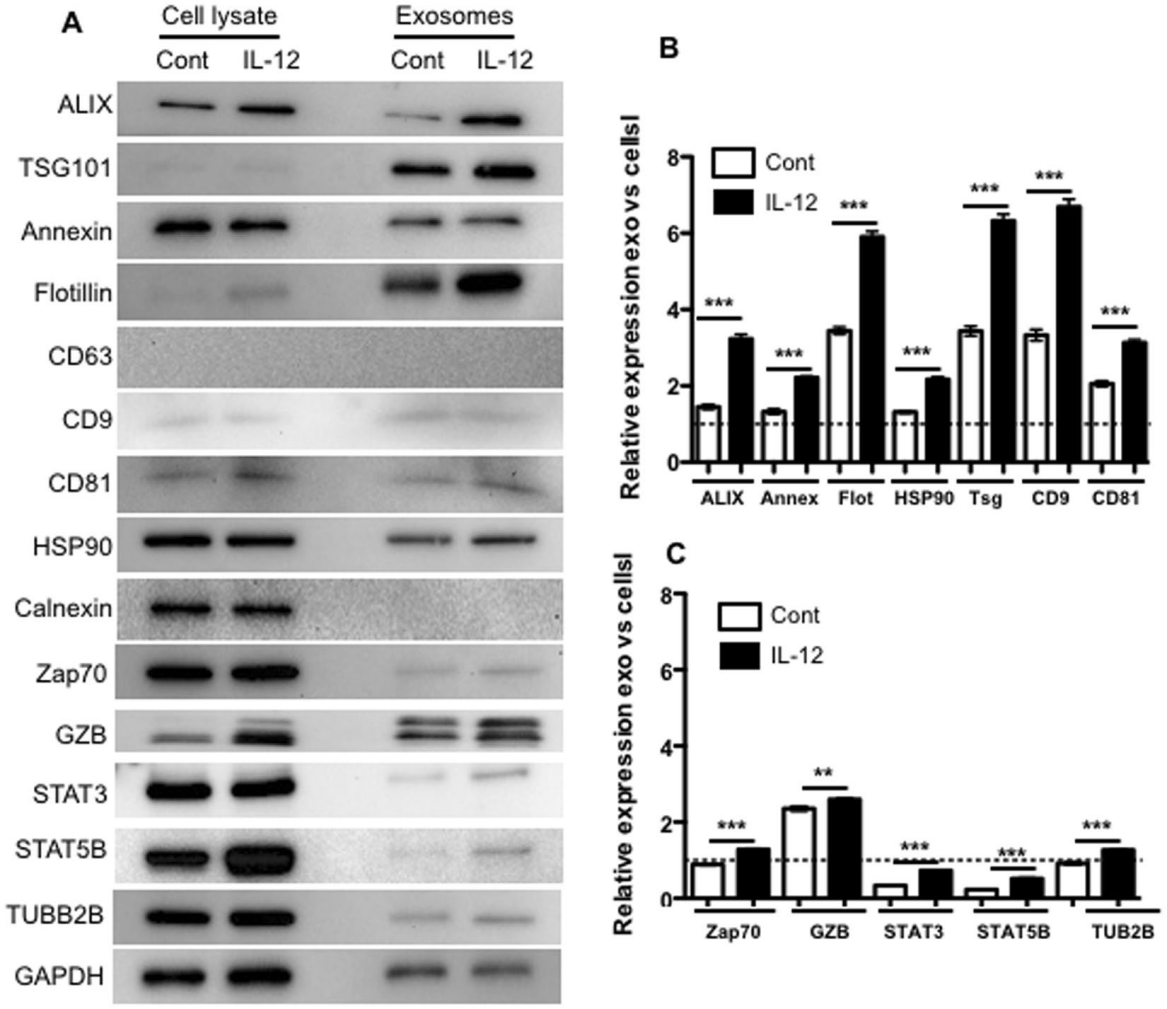
IL-12 stimulation results in selective enrichment of exosomal proteins in activated CTL-derived exosomes. Purified exosomes from 2SI (control) and 3SI (IL-12) activated CTLs were loaded at 10 μg protein in each lane, and processed together for each protein/marker. Uncropped images of the blots for Fig. 2A were shown in Suppl. Figure 3. (**A**) Representative blots. Protein expression levels of (**B**) exosome markers and (**C**) CTL-associated proteins were compared between cells and exosomes from CTLs. Each value is expressed as the relative expression of proteins in exosomes versus cell lysates after normalization to the relevant GAPDH expression level as an internal control. Data are expressed as mean ± SD of three independent experiments. *P < 0.05; **P < 0.01; ***P < 0.001 by unpaired, two-tailed Student’s t-test.

To test whether CTL-derived vesicles contained typical exosome markers^3,33^, equal amounts of protein from purified vesicles and activated CTL lysates were examined. Indeed, both cont-exo and IL-12-exo vesicles expressed exosome markers, such as ALIX, TSG101, and Flotillin-1 (Fig. 2A). Interestingly, CD63, another common exosome marker^33^, was not detectable in either population. The ER protein calnexin was also not detectable in vesicles, consistent with previous reports^3,33–35^. In contrast, activated CTL lysates contained calnexin, but not TSG101 and very low levels of Flotillin (Fig. 2A). Overall, these data support that the vesicles produced by activated CTLs with or without IL-12 stimulation are exosomes.

### IL-12 stimulation results in selective enrichment of proteins in activated CTL exosomes

Upon examination of expression of various protein markers by western blot in cells and exosomes, it became evident that levels of several exosomal proteins were enhanced by IL-12 stimulation (Fig. 2A). These included exosome markers ALIX and CD81, as well as CTL activation-related molecules such as STAT3 and STAT5B (Fig. 2A). To determine which proteins were selectively enhanced in exosomes as compared to their corresponding cells (cell lysate), changes in protein abundances were compared after normalizing to GAPDH levels, which were found to be relatively unaffected by IL-12 stimulation in both cells and exosomes (Suppl. Fig. 1). This comparison revealed significant selective enrichment of all of the exosome markers except CD63 (undetectable) in IL-12-exo vesicles compared with cont-exo ones (Fig. 2B). Further, all of the CTL-related proteins were higher in the IL-12-exo than the cont-exo group, but at similar level as that in whole cell lysate except GZB, which was increased two-fold in vesicles as compared to cell lysate (Fig. 2C). The magnitude of exosomal enrichment for CTL-associated proteins was in general lower than that for exosomal marker proteins (Fig. 2B and C). Interestingly, IL-12 is effective in the induction of effector molecules such as IFNγ in CTLs^36^, but there was no detectable IFNγ in exosomes from either group (data not shown) despite enrichment of GZB levels, suggesting the enrichment of effector-related molecules is also selective, and/or, IFNγ secretion may completely bypass the exosome pathway. GAPDH intensity in cont-exos was about 30% higher than in IL-12-exos, based on the same total protein content (10 μg) (Suppl. Figure 1A). This difference did not change the general conclusion except, although it did affect the GZB data, as indicated in Suppl. Figure 1B,C.

### Exosomes derived from IL-12-conditioned CTLs stimulate bystander CD8^+^ T cells

**Figure 3:**
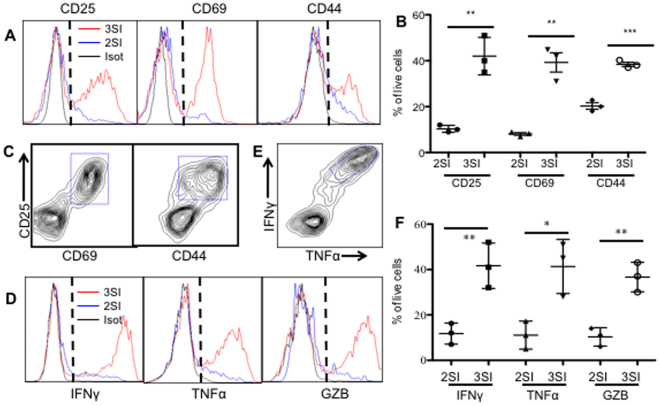
IL-12-conditioned exosomes activate naïve CD8^+^ T cells independent of signaling from TCR. Purified naïve CD8^+^ T cells from B6 mice were cultured with equal amounts of exosomes based on protein concentration and were examined three days after stimulation. (**A** and **D**) Gating strategy for activation markers (**A**) and effector molecules (**D**). Red line: IL-12-exo treated. Blue line: Cont-exo treated. Black line: Isotype control. (**B** and **F**) Quantification of expression of activation markers (**B**) and effector molecules (**F**). (**C** and **E**) Representative dot plots for co-expression of molecules. Results in (**B** and **F**) were expressed as mean + SD, of data from three experiments.

Naïve and effector CD8^+^ T cells can respond to inflammatory stimulation independent of antigen^37,38^. Thus, we hypothesized that activated CTL-derived exosomes may activate naïve CD8^+^ T cells in an antigen-independent manner. To test this, naïve CD8^+^ T cells purified from B6 wild-type mice were cultured directly with exosomes from cont-exo or IL-12-exo. After 72 h, CD25, the α subunit of the IL-2 receptor^39^, was upregulated in ~40% of IL-12-exo-treated CTLs, but far less so (<10%) in cont-exo-treated CTLs (Fig. 3A,B). CD69 and CD44, other CTL activation markers^40^, were similarly upregulated in IL-12-exo-treated CD8^+^ T cells, and less so in cont-exo-treated ones (Fig. 3A and B). Moreover, CD25 and CD69/CD44 were found to be co-expressed in IL-12-exo-treated CD8^+^ T cells (Fig. 3C). Consistent with the upregulation of CTL activation markers shown in Fig. 3A and B, IFNγ, TNFα, and GZB were also upregulated in IL-12-exo-treated CD8^+^ T cells compared to the cont-exo treated (Fig. 3D,E and F). IL-2 could also induce CD8^+^ T cell proliferation independent of TCR signaling both *in vivo* and *in vitro* ^41,42^. To further test the responsiveness of naïve CD8^+^ T cells to IL-2 and IL-12, we purified naïve CD8^+^ T cells from both B6 and OT-I mice and exposed them to these cytokines. More than 90% of the CD8^+^ T cells (from both OT-I and WT B6 animals) died after three days in the presence of IL-2, IL-12, or both. In the few residual, viable cells, CD25, IFNγ, and GZB were induced only by combined IL-2/IL-12, and the enhanced expression of these molecules dropped to background levels when the cytokine concentration was diluted to one-tenth (Suppl. Figure 4 and data not shown). In addition, the pattern of IFNγ and GZB induction (more GZB, less IFNγ) in these IL-12/IL2 conditioned CD8^+^ T cells departed from that of exo-induced cells, wherein IFNγ- and GZB-producing cells were at similar levels. Therefore, we believe that the activation of bystander CD8^+^ T cells induced by IL-12-conditioned exos was unlikely to have been caused by residual cytokines remaining in purified exosomes. These data demonstrate that exosomes derived from fully activated CTLs have the capacity to stimulate naïve CD8^+^ T cells, independent of antigen, in a manner similar to human T cell exosomes^19^.

### IL-12-derived exosomes enhance activation of CTLs under weak antigen stimulation

**Figure 4:**
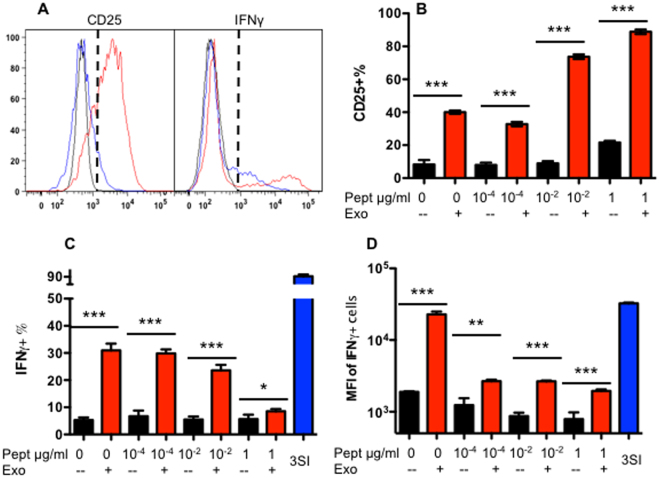
Exosomes derived from 3SI CTLs enhances CTL activation under weak TCR stimulation. Purified naïve CD8^+^ T cells from OT-I mice were cultured for three days with different concentrations of antigen, in the presence or absence of IL-12-exo and/or peptide. (**A**) Gating strategy. Red line: IL-12-exo + antigen. Blue line: Antigen only. Black line: Isotype control. (**B**–**D**) Quantification of expression of CD25 (**B**) and IFNγ (**C** and **D**). Results were expressed as mean + SEM. Each experiment was repeated at least three times with similar results.

CD8^+^ T cells receive different stimulations under different conditions, based on variable availability of antigen, costimulation, and third signal cytokines^43^. To further test if IL-12-derived exosomes could affect CD8^+^ T cells under weak antigen stimulation, naïve OT-I cells were stimulated with SIINFEKL at different concentrations in medium, without costimulation. As expected, CD25 was upregulated weakly, and only at the highest antigen concentrations, (around 20%; black bar in Fig. 4B) (Fig. 4A and B). The addition of IL-12-exo enhanced CD25 expression at all antigen levels, and positively correlated with antigen concentration (Fig. 4B). Conversely, the production of IFNγ was inversely correlated to CD25 expression, gradually declining along with antigen concentration (Fig. 4A and C). This may be due to the drastically enhanced CTL numbers resulting from robust antigen stimulation (data not shown), and suggests that IL-12-exo may contribute to the activation of CD8^+^ T cells under otherwise weak T-cell receptor (TCR) stimulation.

### Exosome secretion may not contribute to memory formation

Memory formation is the gold standard for vaccination, IL-12 is required for memory CTL formation, and IL-12 signaling can be regulated by other regulators, such as rapamycin^30,44,45^. To test the involvement of exosomes in this IL-12-driven memory programing, GW4869, an nSMase2 inhibitor^27,46–50^ used largely *in vitro* rather than *in vivo* ^51^, was added in the presence of IL-12 stimulation for naïve CD8 + T cells^22^. To further test if the presence of IL-12-exo would enhance memory CTL programming, IL-12-exo were added to 2SI stimulated CTLs at the beginning. Three days following stimulation, there were no differences in terms of number or proliferation between GW4869 and control in 3SI stimulation, and neither in 2SI between IL-12-exo treatment and control (data not shown). Cells were then transferred into recipient mice^22^, and the memory programming by 3SI was not affected by the inhibition of exosomes by GW4869, and the IL-12-exo did not enhance memory programming in 2SI stimulated (Suppl. Fig. 2), suggesting that exosomal formation may not be involved in IL-12-driven memory formation.

### Inhibition of exosome formation impairs CTL function during weak stimulation

**Figure 5:**
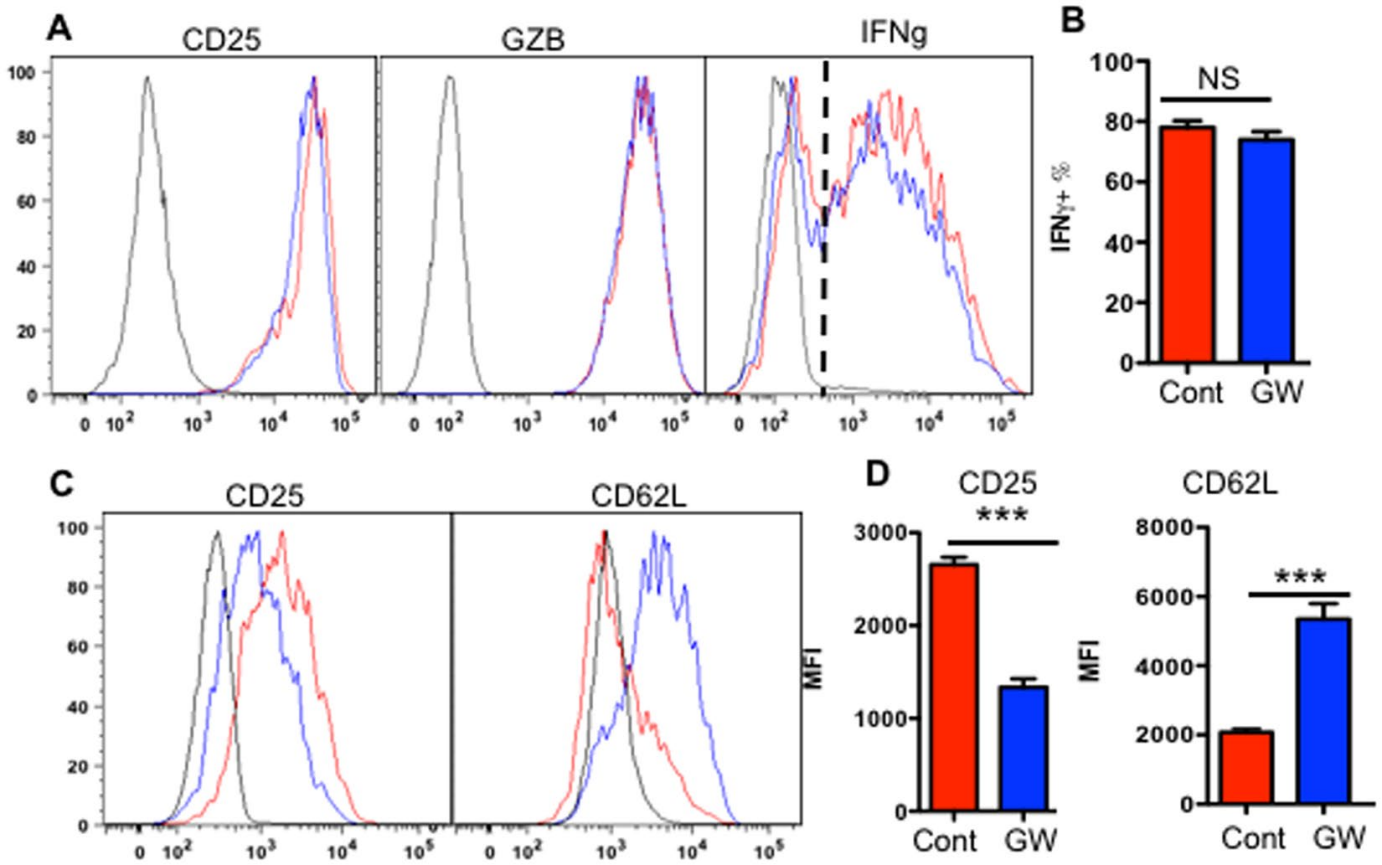
Inhibition of exosome secretion in CTLs does not affect IL-12 induced activation. Purified naïve CD8^+^ T cells from OT-I mice were cultured with the presence (**A** and **B**) or absence (**C** and **D**) of IL-12. Exosome secretion inhibitor GW4869 was tested in each condition. Activation status was examined three days later. (**A** and **C**) Gating strategy for production of effector molecules. Red line: control. Blue line: GW treated. Black line: Isotype control. (**B** and **D)** quantification. Results were expressed as mean + SEM. Each experiment was repeated at least three times with similar results.

In addition to activating bystander CD8^+^ T cells, exosomes might also play a role in intercellular communication during an ongoing CTL response. To investigate if the inhibition of exosome secretion would affect CTL activation, GW4869 was used to suppress exosome secretion. It had no effect on CTLs under IL-12 stimulation, and no changes in IFNγ, GZB, or CD25 levels were observed at normal (Fig. 5A and B) and 1000X diluted antigen concentration (Suppl. Figure 5). However, in the absence of IL-12, GW4869 suppressed CD25 expression (Fig. 5C and D), and CD62L was inhibited by GW4869, suggesting reduced signaling during antigen stimulation (Fig. 5C and D)^21,52^. These data imply that exosomes may be involved in the regulation of CTL activation during weak stimulatory conditions.

### IL-12-induced CTL exosomal proteins are associated with regulatory functions

**Figure 6:**
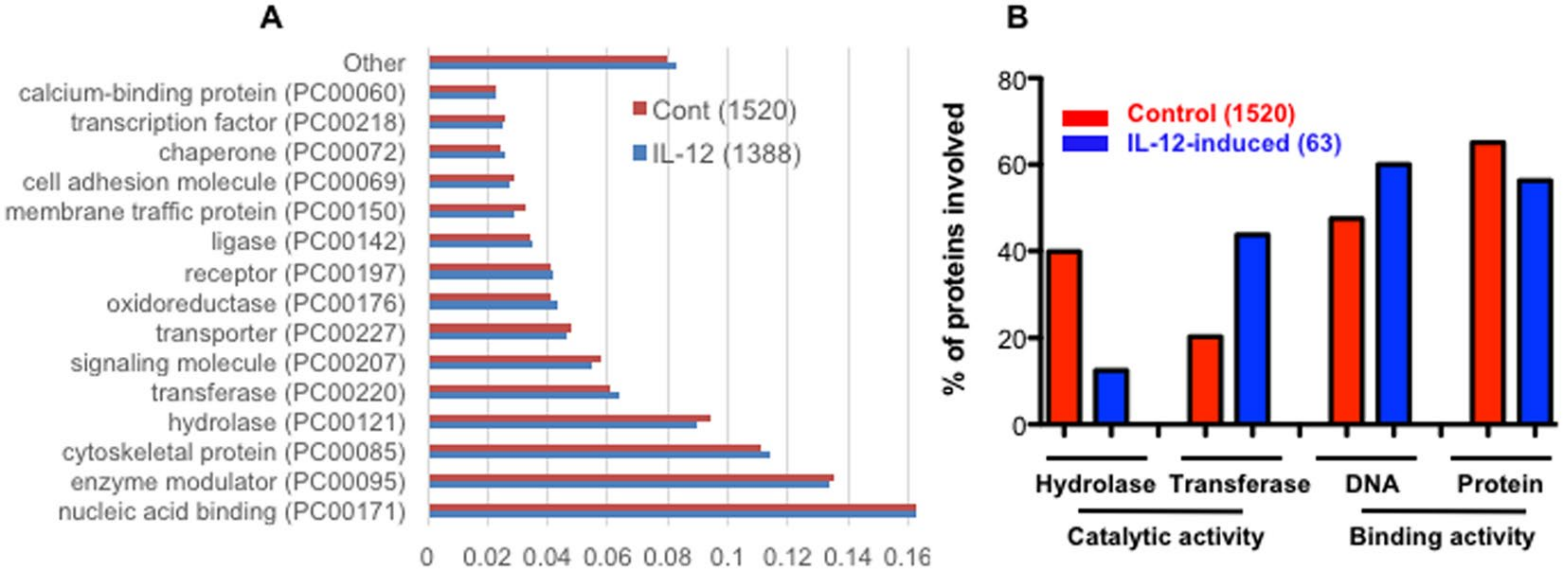
Differential exosomal proteins induced by IL-12. There were 1520 proteins identified by proteomics in control exosomes, and 1388 in IL-12-conditioned exosomes, with very similar protein classification analyzed using PANTHER program (**A**). (**B**) Molecular functions predicted to be associated with IL-12-induced exosomal proteins, which were defined as only present in IL-12 conditioned exosomes compared with control. Numbers in brackets indicates the number of proteins.

To further understand changes in protein composition in activated CTL exosomes based on IL-12 stimulation, proteomic analysis was performed. 1520 protein species were detected in the cont-exo group, while 1388 protein species were detected in the IL-12-exo group. Overall, the protein species were similar in protein classification based on molecular function (Fig. 6A and Suppl. tables 1 and 2). The most abundant protein class was related to nucleic acid binding, followed by enzyme modulators, indicating potential regulatory function for these exosomes. Despite their overall similarities, however, IL-12-stimulated CTL exosomal proteins demonstrated enhanced binding to DNA, but with fewer binding proteins, suggesting their functions may relate to regulating transcription^53–55^. Metabolism related to energy generation usually involves hydrolase and transferase^56–58^; we found a significant number of exosomal proteins classified as hydrolase and transferase in both control and IL-12- stimulated populations (Fig. 6B and Suppl. Tables 2 and 3). However, IL-12 reduced packing of hydrolases in exosomes, and increased transferases (Fig. 6B), which could be one of the possible mechanisms for activation of bystander CD8^+^ T cells. On the other hand, some of the unique proteins in IL-12-induced exosomes fall into typical molecular and cellular functions, including cellular movement, cell morphology, cellular function and maintenance, cell death and survival, and molecular transport (Suppl. Table 4).

## Discussion

Exosomes play significant roles in cell-cell communication in many cell types, including cancer cells, and are known to be produced by stimulated T cells. In our study, we discovered that the features of CTL-derived exosomes are associated with the type of stimulation the CD8^+^ T cells have received. Although 2SI-activated CTLs and 3SI-activated CTLs produce exosomes at similar rates with similar total protein concentrations, the size and specific type of proteins differ noticeably between the two populations. 3SI CTLs produce exosomes containing molecules that can activate naïve bystander CD8^+^ T cells independent of antigen, whereas 2SI CTL-derived exosomes, while larger, have minimal functional capacity. Thus, exosome production may contribute to the host’s ability to mount a robust immune response to one infection while maintaining immunity against others via activation of bystander CD8^+^ T cells through activated CTL-derived exosomes.

Naïve CD8^+^ T cells make up a significant share of immune cells in the body^59^. This population can increase exponentially under extreme conditions, such as in LCMV infection, reaching up to 80% of the total CD8^+^ population in infected animals^60^. To initiate and then sustain this immense CTL population for even a short period of time (7 to 10 days) requires a considerable investment of host resources, including energy and nutrients for CTL proliferation, production of cytokines, and killing of infected cells^61–63^. While there are undoubtedly many factors involved in the maintenance of host innate immunity during a robust immune response, our data indicate that third signal cytokines such as IL-12 may play an important role via regulation of exosome formation and secretion in CTLs. These exosomes can stimulate bystander CD8^+^ T cells without the requirement of antigen stimulation, which may enhance innate immunity by inducing bystander CD8^+^ T cells to produce cytokines, such as IFNγ^64^, in addition to direct destruction of infected cells recognized by these activated bystander CD8^+^ T cells. It would be interesting to determine what effects these exosomes might have on other immune cells, such as dendritic cells.

The effects of exosomes derived from 3SI CTLs may go beyond activation of bystander CD8^+^ T cells. First, these exosomes can boost the activation of CD8^+^ T cells receiving weak TCR stimulation. During real-world infections, the availability of signals to activate CD8^+^ T cells can differ throughout the body^43^, thus, some CD8^+^ T cells may receive less stimulation, and CTL-derived exosomes may help induce a stronger response. Thus, exosomes may play a secondary role during CTL responses by facilitating better intercellular communication between strongly and weakly activated CTLs. Interestingly, this exosome activity is required for CD8^+^ activation in the absence of IL-12 (Fig. 5 and Suppl. Figure 5), suggesting that IL-12-independent exosomes could play an important role in CTL activation, and that IL-12 dependence in immune responses warrants further investigation. Also, the function of exosomes may not necessarily relate to memory programing based on our *in vitro* data, which should be further confirmed *in vivo*. In all, exosomes derived from functional CTLs may play a compensatory role in immune regulation through activation of bystander CD8^+^ T cells and facilitation of intercellular communication during an ongoing CTL response.

It is confusing that the IL-12-exos displayed enhanced CD25 expression but reduced IFNγ production in CTLs as the antigen concentration increased (Fig. 4). There are several possibilities for this discrepancy. First, CD25 expression is associated with IFNγ production, but is more sensitive to exosomes than IFNγ. Antigen stimulation can induce high levels of CD25 expression, but not that of IFNγ^36^. Second, the bystander CD8^+^ T cells that were activated by IL-12-exos (producing IFNγ) might not respond to antigen stimulation, whereas the proliferating CTLs were those that were not affected by IL-12-exos. Therefore, the percentage of these activated bystanders dropped in the expanded population, which increased along with the elevated antigen concentration. Third, it is also possible that as the cell number increases due to proliferation, the number of exosomes available to stimulate individual CD8^+^ T cells becomes smaller. We found the effects of IL-12-exo are dose-dependent (data not shown), and it will be important to evaluate these effects on a physiological range of exosome production *in vivo* in the future.

The variations in CTL exosomes secreted under differential stimulation may be related to exosome biogenesis itself. Exosome formation is driven by two alternate pathways, ESCRT-dependent^9,10^ and ESCRT-independent^3,11,12^. The differences in size and morphology of secreted exosomes suggest that the stimulation strength itself may direct exosome formation and content. The presence of ALIX and TSG101 in both cont-exos and IL-12-exos indicate the exosome formation in CTLs is ESCRT-dependent, and the lack of CD63 further supports this indication, as demonstrated in recent reports^11,65^. However, there were only small differences in exosomal protein expression in CTL lysate between 2SI and 3SI stimulation, indicating that the major effect of IL-12 could be the cargo packing process of exosomes, as significant higher exosomal marker proteins are loaded to exosomes in 3SI CTLs.

Despite the fact that more than 1300 proteins have been identified in both the cont-exo and IL-12-exo groups, only 63 of them were found to be specifically expressed in IL-12-exos. It is possible that only a few proteins are responsible for the activation function of IL-12-exos, which could be tested by gene manipulation in cells or even in mice. It is more likely that these effects are relying on multiple players, as in some metabolic pathways^66,67^. There are many proteins whose abundance was increased in the IL-12-exo versus cont-exo groups which may contribute to the IL-12-exo population’s function. Finally, other players in exosomes may also been involved in immune regulation, such as RNAs, DNA or even some metabolites^3,68,69^. Overall, the protein profile from exosomes derived from CTLs reflect a general regulatory profile on processes related to nucleic acid binding and enzymes, and some of them can be altered by IL-12 during CTL activation.

In summary, in the presence of IL-12, an ongoing CTL immune response may maintain homeostasis of both innate and adaptive immunity through CTL-derived exosomes.

## Methods

### Animals

OT-I mice (gifted by Dr. Mescher, University of Minnesota) possess a transgenic TCR specific for the H-2K^b^ OVA_257–264_ of the ovalbumin protein^22^. All mice were housed in pathogen-free conditions at the University of Maryland, College Park. All animal protocols were reviewed and approved by the Institutional Animal Care and Use Committee (IACUC) at the University of Maryland. All experiments were performed in accordance with relevant guidelines and regulations.

### Naïve T cell purification

This was performed as in previous reports^22^; briefly, inguinal, axillary, brachial, cervical, and mesenteric lymph nodes (LNs) were harvested from WT OT-I or B6 mice, pooled, and homogenized to obtain single cell suspensions. CD8^+^CD44^lo^ cells were enriched by negative selection using MACS magnetic beads (Milteny Biotec, Auburn, CA) wherein cells were coated with FITC-labeled antibodies specific for CD4, B220, I-A^b^, and CD44 (Biolegend San Diego, CA). Anti-FITC magnetic MicroBeads (Miltenyi Biotech) were added, and the suspension passed through separation columns attached to a MACS magnet. Cells that did not bind were collected, and were >95% CD8^+^ and <0.5% CD44^hi^.

### Production of exosomes by CTLs

Naïve OT-I T cells were also purified as described above and stimulated for three days *in vitro* in flat-bottom, microtiter wells coated with antigen (DimerX H-2Kb:Ig fusion protein loaded with OVA_257–264_ peptide) (BD Bioscience, San Jose, CA), and recombinant B7-1/Fc chimeric protein (R&D Systems, Minneapolis, MN) as previously described^22^. 3 × 10^5^ cells in 1.5 mL Allos media were deposited in each well and 2.5 U/mL of IL-2 was added to wells in a 24-well plate. Where indicated, cultures were supplemented with 2 U/mL of murine rIL-12 (R&D Systems). Supernatant was harvested at the end of day three.

### Exosome purification and characterization

To generate exosome-free FBS, FBS (Tissue culture Biologicals, Los Alamitos, CA) was centrifuged at 100,000 *g* for 16 hours to extricate exosomes and Allos medium was prepared using the exosome-free FBS. Supernatant was harvested three days following *in vitro* stimulation. Exosomes from the supernatant were isolated via differential centrifugation, at 300 *g* for 10 min followed by 10,000 *g* for another 10 min to remove cell debris, and then 100,000 *g* for 70 min to pellet exosomes followed by washing with 1XPBS and further pelleting^25,27,70^. Exosome pellets were resuspended in 1XPBS and divided into small aliquots to avoid repeated thaw-freeze cycles. Protein concentrations of the exosome preparations were determined by the Bradford microassay method (Bio-Rad Laboratories)^27^. Size distribution of exosomes was assessed using a Malvern Zetasizer Nano ZS90. Exosome concentration was measured by nanoparticle tracking analysis (NTA) using a NanoSight LM10. Purified exosomes were diluted twenty-fold in 400 µL of PBS prior to loading into the NanoSight analysis chamber; data were collected from three different fields of view per sample (1 min each; at least 100 particle tracks) and analyzed by NTA2.1 software. Measurements were independently replicated two additional times for a total n = 3.

### Exosome function

Naïve CD8^+^ T cells from B6 or OT-I mice were cultured with equal amounts of purified exosomes based on equivalent exosomal protein quantities (33 µg/mL in medium), as during human T cell stimulation^19^. The medium is identical to that used in CTL culture^71^, excepting the addition of IL-2^22,29,30^. GW4869, an nSMase2 inhibitor^27,46–50^, was purchased from Cayman Chemicals (Ann Arbor, MI) and added at the beginning of culture at 2 μM.

### Western Blot

Ten μg of protein from cell lysate or purified exosomes was separated by electrophoresis on 10% SDS-polyacrylamide gel and transferred to a Polyvinylidene Difluoride (PVDF) membrane (BioRad, Hercules, CA). The membrane was blocked with a 20 mM Tris-HCl, pH 7.6, 150 mM NaCl, and 0.05% Tween-20 blocking solution (TBST) containing 1% bovine serum albumin (BSA) and then incubated with the first antibody at room temperature (RT) for 1 h. After washing in TBST 3 times, the membrane was incubated with horseradish peroxidase (HRP)-conjugated anti-bovine IgG secondary antibody for 1 h at RT. Signals were detected using the SuperSignal West Pico Chemiluminescent Substrate (Thermo Scientific, Waltham, MA) and a Gel Doc imaging system (BioRad). All antibodies were from Cell Signal Technology, Danvers, MA), Bio-Rad, and Biolegend.

### Intracellular cytokine staining

For IFNγ induction, following 3 days of *in vitro* stimulation, cells were incubated in RP-10 with 0.2 µM OVA_257–264_ peptide and 1 µL Brefeldin A (Biolegend) for 3.5 hrs at 37 °C. For IFNγ and GZB staining, cells were fixed in fixing buffer (Biolegend) for 15 min at 4 °C, permeabilized in saponin-containing Perm/Wash buffer (Biolegend) for another 15 min at 4 °C, and then stained with PE-conjugated antibody to IFNγ or GZB for 30 min at 4 °C. Cells were then washed once with Perm/Wash buffer, and once with PBS containing 2% FBS.

### Transmission Electron Microscopy

Exosomes containing 0.03–0.3 pg protein were suspended in 2% glutaraldehyde and applied to a Formvar-coated grid and negatively stained with uranyl acetate. Electron micrographs were obtained using a Zeiss EM10 transmission electron microscope at an accelerating voltage of 80keV^26^.

### Proteomics

Exosomes were suspended in 8 M urea and incubated at 4 °C two disrupt their membranes. *Tryptic digestion:* Exosomal proteins were reduced with DTT, alkylated with iodoacetamide, and digested with 2 µg Trypsin/LysC Mix (Promega, Madison, WI) at 35 °C, first at 4 M for 1 hr, then further diluted to 0.8 M urea to activate the trypsin, and incubated overnight. Tryptic digests were acidified with 2 µL TFA and desalted with C18 TopTip (Glygen Corp., Columbia, MD). Eluted peptides were vacuum-dried and dissolved in 50 µL solvent A. Concentration was estimated using a Qubit 3.0 Fluorometer. LCMSMS analysis was carried out using a Dionex U3000 nanoHPLC system interfaced to a Thermo Scientific orbitrap Fusion Lumos mass spectrometer. Each sample was analyzed three times. For each analysis, 1 µg of tryptic digest was injected into an Accalaim™ PepMap™ 100 trap column, (5 µm, 100 Å, 300 µm × 5 mm) and desalted at 5 µL/min with 100% Solvent A (2.5% ACN, 0.1% formic acid) for 5 min. The peptides were then separated with an Accalaim PepMap™ 100 nano column (3 µm, 100 Å, 75 µm × 250 mm) using a linear gradient of 2–52% Solvent B (75% ACN, 0.1% formic acid) over 160 min. Precursor masses were detected in the Orbitrap at R = 120,000 (m/z 200). Fragment masses were detected in linear ion trap at unit mass resolution. Data dependent MSMS was carried out at a cycle time of 3 sec. Dynamic exclusion was at 30 sec. Raw data was searched against the Uniprot mouse proteome (uniprot.org) via Proteome Discoverer 2.1 using Sequest HT and Mascot search engines. M oxidation and NQ deamidation were set as variable modifications, and carbomidomethylation of C was set as the fixed modification. Precursor mass tolerance was 20 ppm, later filtered to 5 ppm in the report. Fragment mass tolerance was 0.6 Da. Search results were parsed into Scaffold (Proteome Software, Portland, OR) for validation and to eliminate redundancy. Proteins with significantly different abundances (p < 0.05) across the three replications were identified using ANOVA on total spectra count.

### Enriched pathway analysis of differentially expressed genes

Data were uploaded into the Ingenuity Pathways Analysis (IPA) software (Ingenuity Systems, http://www.ingenuity.com). The IPA database is maintained and edited by humans, and contains genes, proteins, and RNA in order to not only find associations between expression data and canonical pathways, but also build new networks. The significance of associations was computed using the right-tailed Fisher exact test. All signaling pathways identified by IPA with a *P* value ≤ 0.05 have a statistically significant, nonrandom association.

### Statistical Analysis

We used an unpaired, two-tailed Student’s t-test in GraphPad (Prism 5.0 software; GraphPad Prism, La Jolla, CA, USA) for statistical analysis of significance.

## Electronic supplementary material

Supplementary Information
