# Supplementary material for: IL-12 stimulates CTLs to secrete exosomes capable of activating bystander CD8+ T cells
Source: Sci Rep. 2017 Oct 17;7:13365. doi: 10.1038/s41598-017-14000-z (PMC5645350; doi:10.1038/s41598-017-14000-z)
Supplement: Supplementary file 1 — Supplementary Information [file 41598_2017_14000_MOESM1_ESM.pdf]

IL-12 stimulates CTLs to secrete exosomes capable of activating bystander CD8^{+} T cells

Lei Li[1], Steven M. Jay[2], Yan Wang[3], Shu-Wei Wu[1] and Zhengguo Xiao[1]

[1]Department of Animal and Avian Sciences, University of Maryland, College Park, Maryland 20742, USA;

[2]Fischell Department of Bioengineering, University of Maryland, College Park, Maryland 20742, USA;

[3]Department of Cell Biology and Molecular Genetics, University of Maryland, College Park, Maryland 20742, USA;

Corresponding Author: Zhengguo Xiao, Ph.D.
Department of Animal and Avian Sciences
University of Maryland
College Park, MD 20742 USA
Phone: 301-405-6258
FAX: 301-405-7980
Email: xiao0028@umd.edu

Index for supplementary information

Information	Pages
Suppl.Fig.1 Comparison of GAPDP expression	1
Suppl.Fig.2 Memory CTL programming is not affected by exosomes.	1
Suppl.Fig.3 Uncropped images of the blots for Fig.2a	2
Suppl.Fig.4 Response of naïve CD8^{+} T cells to cytokines	3
Suppl.Fig.5 Effects of exosome inhibition on CD8^{+} T cells under low antigen concentration	3
Suppl.table.1 Proteins Detected in Exosomes	4-43
Suppl.table.2 PANTHER Analysis of Exosomal Proteins	44-45
Suppl.table.3 Unique Proteins Induced by IL-12	46-47
Suppl.table.4 Molecular and Cellular Functions of IL-12-induced Unique Proteins	48

Supplementary information

Manuscript: IL-12 stimulates CTLs to secrete exosomes capable of activating bystander CD8$^+$ T cells

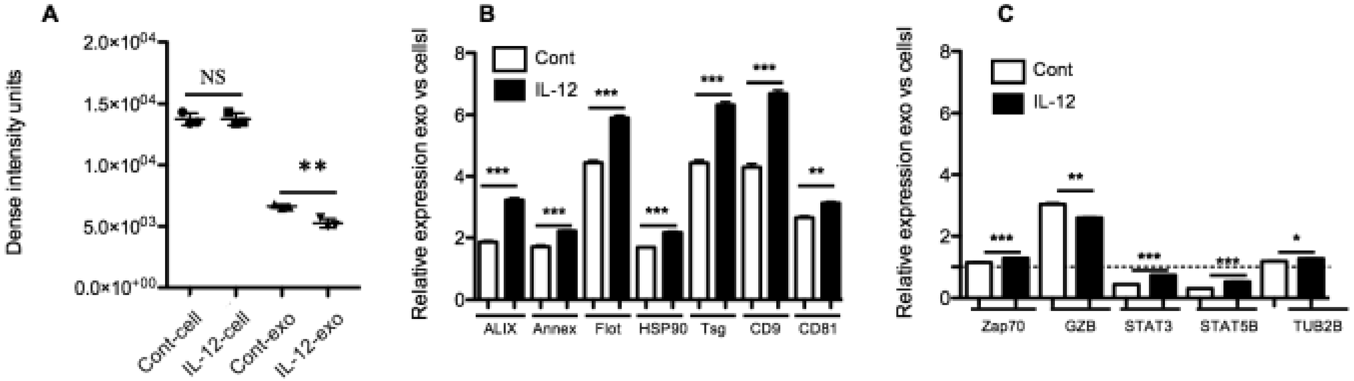

Supplementary Fig.1. Equal amount of protein (10 μg) from either cell lysate or exosomes was examined in western blot. A. GAPDH was quantified based on blot intensity. B-C. Reanalysis of the same data as in Fig.2 B-C, adjusted by the difference of the mean value of GAPDH between cont-exo and IL-12-exo, so the protein level in cont-exo was increased by about 30%, whereas protein level in IL-12-exo was kept as unchanged.

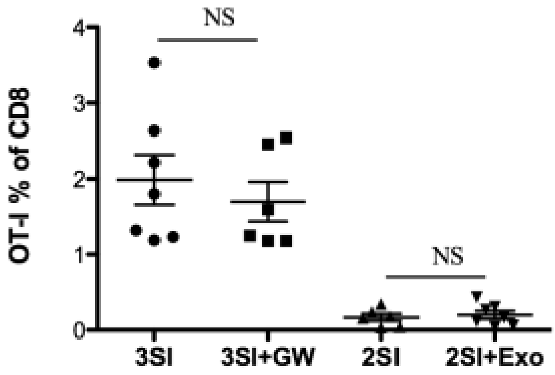

Supplementary Fig.2. Naïve OT-I cells were cultured with either 3SI (including IL-12) or 2SI for three days. GM: GW4869, at 2 μM at the beginning of stimulation. Exo: IL-12-conditioned exosomes, at 33 μg/ml of protein concentration, added at the beginning of stimulation. CTLs were washed extensively, and were transferred into naïve B6 mice at $2X10^5$/mouse through i.v. Blood samples was drawn at day 15 after transfer to detect the presence of OT-I cells.

Supplementary Figure 3. Uncropped images of the blots for Fig.2a

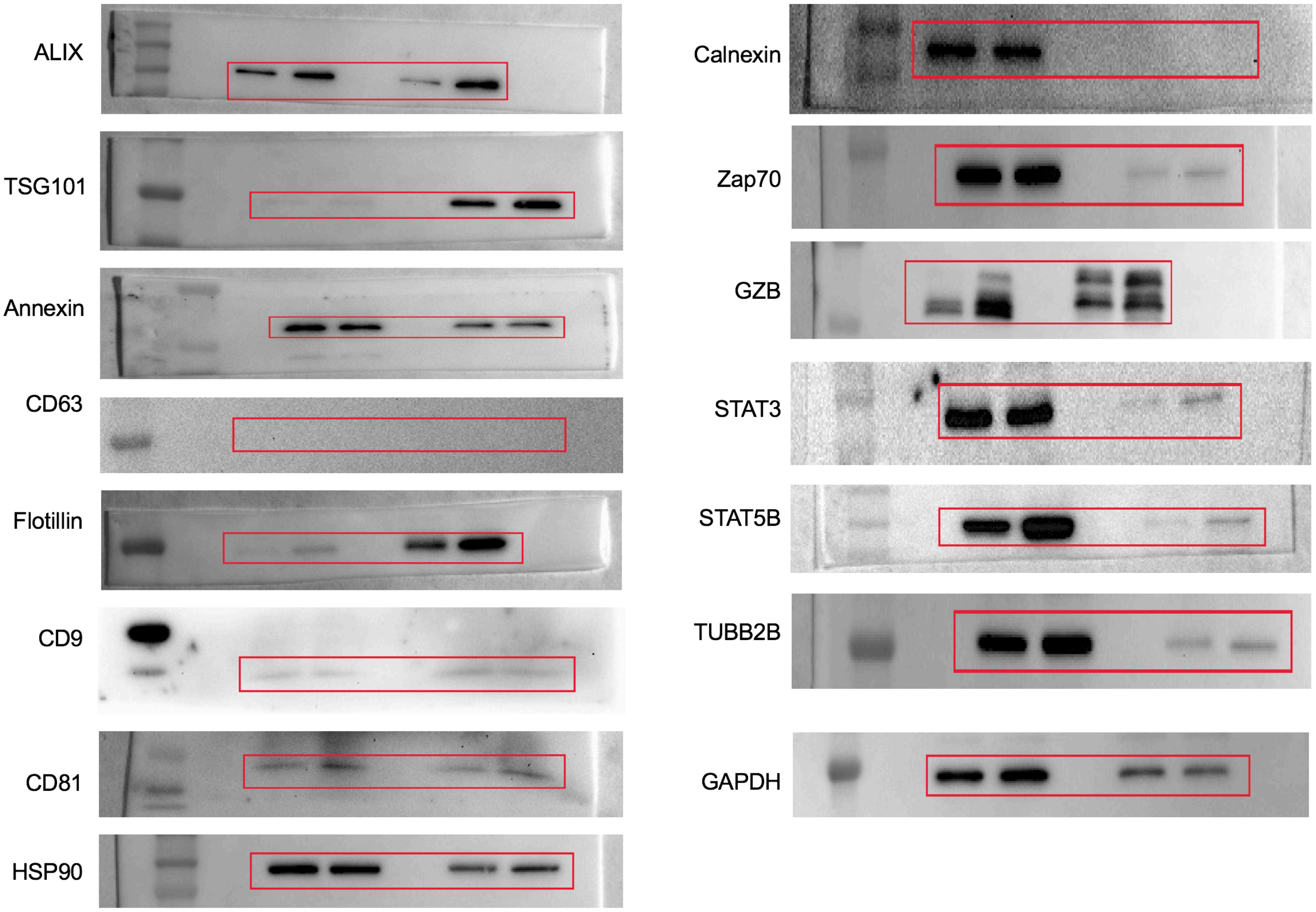

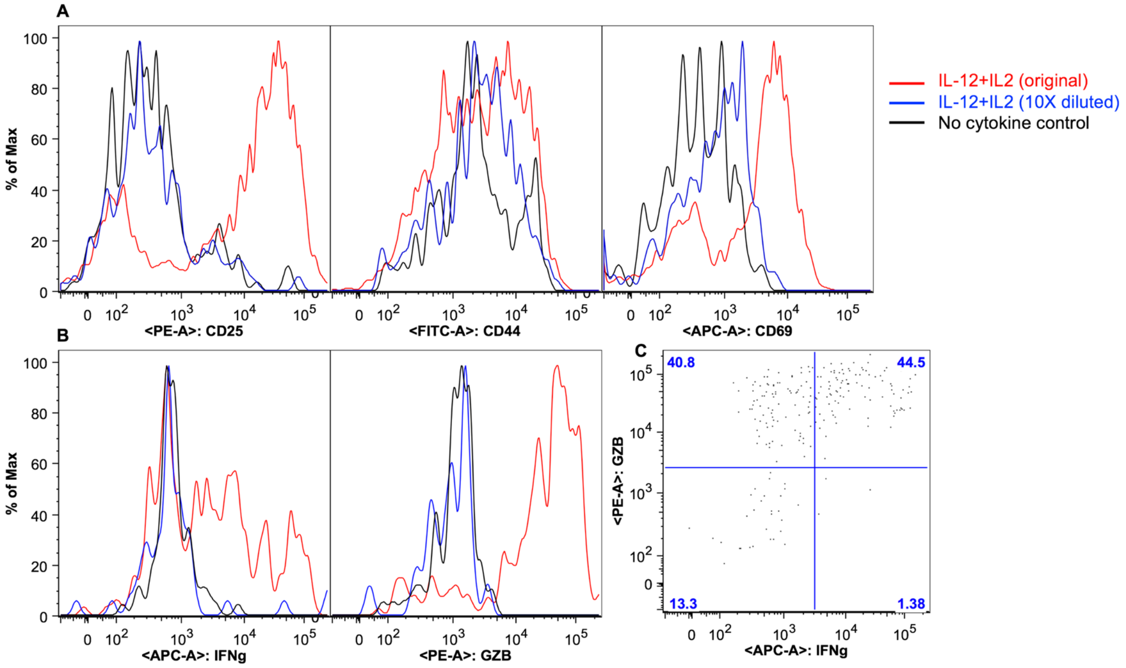

Supplementary Fig.4. Naïve CD8$^+$ T cells purified from B6 were cultured with IL-2 and/or IL12 for three days. Viable cells were analyzed for expression of CD25, CD44 and CD69 (A), and IFNγ/GZB (B). C. A representative dot plot from IL-12/IL2 conditioned CD8$^+$ T cells. This shows all the detected cells (about 300) from the initial input about 0.5X10^5 naïve CD8$^+$ T cells. The experiment was performed twice on naïve CD8$^+$ T cells from both B6 and OT-I mice, with similar results.

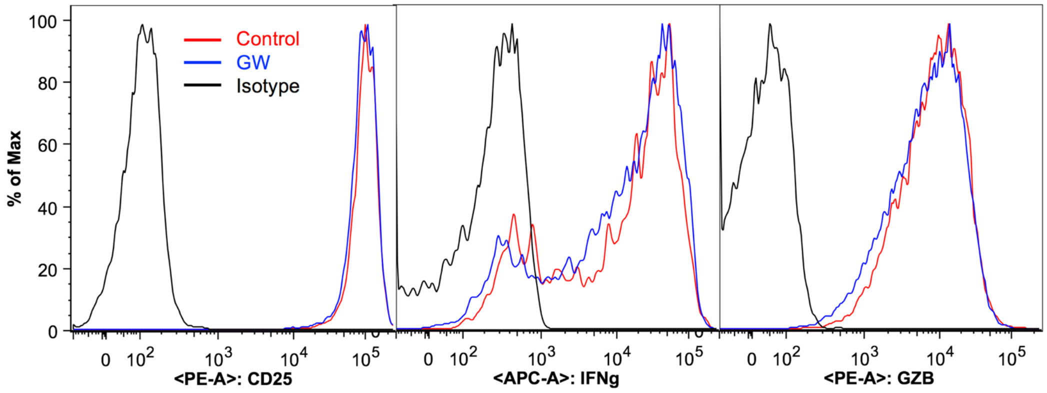

Supplementary Fig.5. Naïve OT-I CD8$^+$ cells were cultured for three days with 3SI (including IL-12), but with antigen diluted at 1000X based on the standard concentration used in Fig.5, in the presence (GW) or absence (Control) of GW. The experiment was tested twice with similar results.

Supplementary table-1 Proteins Detected in Exosomes

	ID	Protein name	2SI	3SI	Numbers indi
702	P60710	Actin, cytoplasmic 1 OS=Mus musculus (	437	424	
1477	Q4KL81	Actin, cytoplasmic 2 OS=Mus musculus (	427	416	
574	P26041	Moesin OS=Mus musculus GN=Msn PE=	229	186	
573	P26039	Talin-1 OS=Mus musculus GN=Tln1 PE=	156	134	
777	P68134	Actin, alpha skeletal muscle OS=Mus mus	146	148	
474	P06800	Receptor-type tyrosine-protein phosphatas	146	120	
2563	S4R1M0	Receptor-type tyrosine-protein phosphatas	146	120	
2564	S4R1S4	Receptor-type tyrosine-protein phosphatas	146	120	
2566	S4R2V1	Protein tyrosine phosphatase, receptor typ	146	120	
760	P63017	Heat shock cognate 71 kDa protein OS=M	128	103	
549	P18760	Cofilin-1 OS=Mus musculus GN=Cfl1 PE	112	96	
1018	Q3TJX0	Putative uncharacterized protein OS=Mus	111	103	
1663	Q61233	Plastin-2 OS=Mus musculus GN=Lcp1 PI	111	103	
1478	Q4KML7	Ezrin OS=Mus musculus GN=Ezr PE=1 S	111	84	
240	E9Q5G3	Kinesin-like protein KIF23 OS=Mus musc	109	76	
2155	Q8VDN2	Sodium/potassium-transporting ATPase su	108	90	
2457	Q9JKF1	Ras GTPase-activating-like protein IQGA	107	117	
575	P26043	Radixin OS=Mus musculus GN=Rdx PE=	104	84	
128	B7FAU9	Filamin, alpha OS=Mus musculus GN=Fl	99	88	
1971	Q8BTM8	Filamin-A OS=Mus musculus GN=Flna P	99	88	
823	P99024	Tubulin beta-5 chain OS=Mus musculus (	98	87	
1371	Q3ULT2	Actinin alpha 4 OS=Mus musculus GN=A	97	98	
502	P11499	Heat shock protein HSP 90-beta OS=Mus	94	93	
2152	Q8VDD5	Myosin-9 OS=Mus musculus GN=Myh9 I	91	73	
534	P16858	Glyceraldehyde-3-phosphate dehydrogena	86	83	
780	P68372	Tubulin beta-4B chain OS=Mus musculus	86	74	
1292	Q3UDK4	Annexin OS=Mus musculus GN=Anxa6 I	84	77	
697	P58252	Elongation factor 2 OS=Mus musculus GI	83	85	
1605	Q5SUA5	Unconventional myosin-Ig OS=Mus musc	83	83	
312	F8WIT2	Annexin OS=Mus musculus GN=Anxa6 I	83	76	
1616	Q5SXR6	Clathrin heavy chain OS=Mus musculus (	82	98	
1723	Q68FD5	Clathrin heavy chain 1 OS=Mus musculus	82	98	
1696	Q62351	Transferrin receptor protein 1 OS=Mus m	82	79	
1897	Q8BFZ3	Beta-actin-like protein 2 OS=Mus muscul	80	82	
679	P52480	Pyruvate kinase PKM OS=Mus musculus	79	84	
666	P49025-5	Isoform 5 of Citron Rho-interacting kinas	78	34	
504	P11835	Integrin beta-2 OS=Mus musculus GN=It	75	60	

138	B7ZN90	Itgal protein OS=Mus musculus GN=Itgal	73	58
242	E9Q5M7	Integrin alpha-L OS=Mus musculus GN=I	73	58
276	E9QNL8	Integrin alpha-L OS=Mus musculus GN=I	73	58
351	H2ELL3	Alpha L integrin (Fragment) OS=Mus mu	73	58
885	Q3T9N8	Putative uncharacterized protein OS=Mus	73	58
903	Q3TB85	Putative uncharacterized protein OS=Mus	73	58
1140	Q3U159	Itgal protein OS=Mus musculus GN=Itgal	73	58
2482	Q9QYB1	Chloride intracellular channel protein 4 O	72	56
271	E9QM99	Dedicator of cytokinesis protein 10 OS=M	67	62
1322	Q3UH60	Disco-interacting protein 2 homolog B OS	66	46
468	P05213	Tubulin alpha-1B chain OS=Mus musculu	65	57
1568	Q5FWB7	Fructose-bisphosphate aldolase OS=Mus r	64	70
779	P68369	Tubulin alpha-1A chain OS=Mus musculu	64	55
550	P19096	Fatty acid synthase OS=Mus musculus GN	64	52
2533	Q9WVK4	EH domain-containing protein 1 OS=Mus	63	72
1879	Q80Y52	Heat shock protein 90, alpha (Cytosolic),	63	66
126	B2RY84	Kinesin-like protein OS=Mus musculus G	61	42
376	L0N7N1	Kinesin-like protein KIF14 OS=Mus musc	61	42
1840	Q7TPR4	Alpha-actinin-1 OS=Mus musculus GN=A	59	48
778	P68368	Tubulin alpha-4A chain OS=Mus musculu	57	49
486	P09411	Phosphoglycerate kinase 1 OS=Mus musc	56	59
1878	Q80Y09	Pdcd6ip protein OS=Mus musculus GN=P	56	48
2521	Q9WU78	Programmed cell death 6-interacting prote	56	48
482	P08752	Guanine nucleotide-binding protein G(i) s	54	48
1714	Q64478	Histone H2B type 1-H OS=Mus musculus	54	43
2238	Q99JI6	Ras-related protein Rap-1b OS=Mus musc	53	44
1390	Q3UP78	T-cell surface glycoprotein CD5 OS=Mus	50	48
2189	Q91X69	CD5 antigen OS=Mus musculus GN=Cd5	50	48
471	P06240	Proto-oncogene tyrosine-protein kinase LC	50	43
2552	Q9Z1Q5	Chloride intracellular channel protein 1 O	49	57
439	O88342	WD repeat-containing protein 1 OS=Mus	49	52
791	P70441	Na(+)/H(+) exchange regulatory cofactor	49	46
2534	Q9WVM1	Rac GTPase-activating protein 1 OS=Mus	49	37
1548	Q58E70	Tpm3 protein OS=Mus musculus GN=Tp	48	55
1311	Q3UFR4	Amino acid transporter OS=Mus musculu	47	41
2433	Q9ESU7	Amino acid transporter OS=Mus musculu	47	41
951	Q3TH01	H-2 class I histocompatibility antigen, K-I	46	46
2570	W5XHY0	MHC class I antigen OS=Mus musculus C	46	46
500	P11276	Fibronectin OS=Mus musculus GN=Fn1 I	46	34
1320	Q3UGY5	Putative uncharacterized protein OS=Mus	46	34

495	P10852-2	Isoform 2 of 4F2 cell-surface antigen heav	45	48
831	Q01853	Transitional endoplasmic reticulum ATPa	44	50
743	P62835	Ras-related protein Rap-1A OS=Mus mus	43	39
505	P11983	T-complex protein 1 subunit alpha OS=M	43	35
1067	Q3TQ70	Beta1 subnuit of GTP-binding protein OS	42	40
768	P63101	14-3-3 protein zeta/delta OS=Mus muscul	42	34
538	P17742	Peptidyl-prolyl cis-trans isomerase A OS=	41	49
922	Q3TE63	Peptidyl-prolyl cis-trans isomerase OS=M	41	49
1734	Q6A0F1	MKIAA0002 protein (Fragment) OS=Mu	41	41
630	P40124	Adenylyl cyclase-associated protein 1 OS	41	36
424	O55098	Serine/threonine-protein kinase 10 OS=M	41	20
1011	Q3TJH1	Putative uncharacterized protein OS=Mus	40	40
2405	Q9DC51	Guanine nucleotide-binding protein G(k)	40	40
1509	Q542X7	Chaperonin subunit 2 (Beta), isoform CR	40	38
461	P01899	H-2 class I histocompatibility antigen, D-	39	37
528	P15702	Leukosialin OS=Mus musculus GN=Spn	39	37
1745	Q6KAS9	MFLJ00087 protein (Fragment) OS=Mus	38	36
1438	Q3UZQ3	Elongation factor 1-alpha OS=Mus muscu	38	34
798	P80315	T-complex protein 1 subunit delta OS=M	38	33
987	Q3TII0	T-complex protein 1 subunit delta OS=M	38	33
1603	Q5SS40	14-3-3 protein epsilon OS=Mus musculus	38	30
2086	Q8CGC7	Bifunctional glutamate/proline--tRNA lig	38	27
2515	Q9WTI7	Unconventional myosin-Ic OS=Mus musc	38	22
1128	Q3TZH4	Granzyme B OS=Mus musculus GN=Gzn	37	54
1144	Q3U1N0	Coronin OS=Mus musculus GN=Coro1a	37	41
1148	Q3U232	Coronin OS=Mus musculus GN=Coro1a	37	41
470	P06151	L-lactate dehydrogenase A chain OS=Mu	37	36
2091	Q8CGP5	Histone H2A type 1-F OS=Mus musculus	37	35
950	Q3TGW0	Putative uncharacterized protein OS=Mus	37	34
799	P80316	T-complex protein 1 subunit epsilon OS=	37	33
2047	Q8C605	ATP-dependent 6-phosphofructokinase O	37	32
2522	Q9WUA3	ATP-dependent 6-phosphofructokinase, p	37	32
554	P20029	78 kDa glucose-regulated protein OS=Mu	36	37
1232	Q3U9G2	Putative uncharacterized protein OS=Mus	36	37
1651	Q61003	T-cell differentiation antigen CD6 OS=M	36	29
2184	Q91WN5	CD6 antigen OS=Mus musculus GN=Cd6	36	29
391	O08992	Syntenin-1 OS=Mus musculus GN=Sdcb	36	28
443	O88601	Syntenin OS=Mus musculus GN=Sdcbp	36	28
928	Q3TET7	Putative uncharacterized protein OS=Mus	36	28
1052	Q3TMX0	MCG4375, isoform CRA_b OS=Mus mu	36	28

1225	Q3U902	Putative uncharacterized protein OS=Mus	36	28
1264	Q3UBE4	Putative uncharacterized protein OS=Mus	36	28
1274	Q3UC68	Putative uncharacterized protein OS=Mus	36	28
750	P62880	Guanine nucleotide-binding protein G(I)/(	35	34
149	B9EHN0	Ubiquitin-activating enzyme E1, Chr X O	34	39
1683	Q61790	Lymphocyte activation gene 3 protein OS	34	35
1150	Q3U2G2	Heat shock 70 kDa protein 4 OS=Mus mu	34	33
1544	Q571M2	MKIAA4025 protein (Fragment) OS=Mu	34	33
872	Q1MWP9	EH-domain containing 4 (Fragment) OS=	34	32
1043	Q3TM70	EH domain-containing protein 4 OS=Mus	34	32
116	B2RUE8	Map4k4 protein OS=Mus musculus GN=l	34	26
2342	Q9CZI7	Annexin OS=Mus musculus GN=Anxa2 I	33	30
2531	Q9WVA4	Transgelin-2 OS=Mus musculus GN=Tag	32	32
1669	Q61598	Rab GDP dissociation inhibitor beta OS=l	32	29
782	P70168	Importin subunit beta-1 OS=Mus musculu	32	26
938	Q3TFE8	Putative uncharacterized protein OS=Mus	32	26
1851	Q7TSZ6	Karyopherin (Importin) beta 1 OS=Mus n	32	26
605	P32037	Solute carrier family 2, facilitated glucose	32	23
1462	Q4FJP8	Slc2a3 protein OS=Mus musculus GN=Sl	32	23
1938	Q8BLF7	Putative uncharacterized protein OS=Mus	32	23
314	F8WIX8	Histone H2A OS=Mus musculus GN=His	31	31
40	A2APM2	CD44 antigen OS=Mus musculus GN=Cd	31	29
526	P15379	CD44 antigen OS=Mus musculus GN=Cd	31	29
2107	Q8K1B8	Fermitin family homolog 3 OS=Mus mus	31	26
119	B2RUR3	ATP-binding cassette, sub-family B (MDI	31	25
585	P27659	60S ribosomal protein L3 OS=Mus muscu	31	24
1258	Q3UB15	Putative uncharacterized protein OS=Mus	31	24
1260	Q3UB90	Putative uncharacterized protein OS=Mus	31	24
153	B9EKE9	Ddx3x protein OS=Mus musculus GN=D	31	23
1068	Q3TQX5	ATP-dependent RNA helicase DDX3X O	31	23
2303	Q9CVB6	Actin-related protein 2/3 complex subunit	31	20
425	O55107	Basigin (Fragment) OS=Mus musculus G	30	27
963	Q3THH8	Putative uncharacterized protein OS=Mus	30	22
704	P60843	Eukaryotic initiation factor 4A-I OS=Mus	29	27
1475	Q4FZL1	Eif4a1 protein (Fragment) OS=Mus musc	29	27
1166	Q3U4U6	T-complex protein 1 subunit gamma OS=	29	26
906	Q3TCE7	D-3-phosphoglycerate dehydrogenase OS	29	25
1681	Q61753	Putative uncharacterized protein OS=Mus	29	25
2202	Q91Z25	Actin-related protein 2/3 complex subunit	29	25
2528	Q9WV32	Actin-related protein 2/3 complex subunit	29	25

1589	Q5PPQ7	Coronin OS=Mus musculus GN=Coro1c l	28	25
1719	Q64737	Trifunctional purine biosynthetic protein a	28	25
2523	Q9WUM4	Coronin-1C OS=Mus musculus GN=Coro	28	25
755	P62962	Profilin-1 OS=Mus musculus GN=Pfn1 P	28	24
458	P01831	Thy-1 membrane glycoprotein OS=Mus m	28	23
1498	Q53YX2	CD90.1 OS=Mus musculus GN=Thy1 PE	28	23
1697	Q62418	Drebrin-like protein OS=Mus musculus G	28	23
539	P17751	Triosephosphate isomerase OS=Mus musc	27	32
532	P16546	Spectrin alpha chain, non-erythrocytic 1 C	27	14
724	P62137	Serine/threonine-protein phosphatase PP1	26	26
110	B2RQC6	CAD protein OS=Mus musculus GN=Cad	26	25
257	E9QAI5	CAD protein OS=Mus musculus GN=Cad	26	25
811	P97370	Sodium/potassium-transporting ATPase su	26	22
473	P06745	Glucose-6-phosphate isomerase OS=Mus	26	19
228	E9Q3L2	Protein Pi4ka OS=Mus musculus GN=Pi4	26	11
1739	Q6DIC7	Phosphatidylinositol 4-kinase, catalytic, al	26	11
1595	Q5RKN9	Capping protein (Actin filament) muscle Z	25	23
1534	Q564E8	60S ribosomal protein L4 OS=Mus muscu	25	21
1752	Q6NZB6	Ly9 protein (Fragment) OS=Mus musculu	25	21
597	P29351	Tyrosine-protein phosphatase non-recepto	25	19
1276	Q3UCJ0	Tyrosine-protein phosphatase non-recepto	25	19
1602	Q5SQX6	Cytoplasmic FMR1-interacting protein 2 (	25	19
509	P12970	60S ribosomal protein L7a OS=Mus musc	25	16
1562	Q5EBG5	Ribosomal protein L7A OS=Mus musculu	25	16
1756	Q6P1A9	Ribosomal protein L7A OS=Mus musculu	25	16
1867	Q80UT7	Rpl7a protein (Fragment) OS=Mus muscu	25	16
1486	Q4VAE6	Ras family member A OS=Mus musculus	24	23
646	P43404	Tyrosine-protein kinase ZAP-70 OS=Mus	24	22
1531	Q549Q4	CD2 antigen OS=Mus musculus GN=Cd2	24	22
781	P68510	14-3-3 protein eta OS=Mus musculus GN	24	20
863	Q149Z9	Histone H1.3 OS=Mus musculus GN=His	24	14
754	P62960	Nuclease-sensitive element-binding protei	23	32
2397	Q9DBJ1	Phosphoglycerate mutase 1 OS=Mus musc	23	26
765	P63087-2	Isoform Gamma-2 of Serine/threonine-pro	23	23
1202	Q3U7K1	Serine/threonine-protein phosphatase OS=	23	23
1800	Q6ZWM8	Serine/threonine-protein phosphatase OS=	23	23
2110	Q8K1X4	NCK associated protein 1 like OS=Mus m	23	19
583	P27601	Guanine nucleotide-binding protein subun	23	16
596	P29341	Polyadenylate-binding protein 1 OS=Mus	23	15
1222	Q3U8U8	Polyadenylate-binding protein OS=Mus m	23	15

2413	Q9DCL9	Multifunctional protein ADE2 OS=Mus m	23	14
2404	Q9DC42	Uncharacterized protein OS=Mus muscul	23	11
2480	Q9QXY6	EH domain-containing protein 3 OS=Mus	22	24
680	P53986	Monocarboxylate transporter 1 OS=Mus r	22	23
1073	Q3TRK9	Putative uncharacterized protein OS=Mus	22	23
1955	Q8BPS5	Putative uncharacterized protein OS=Mus	22	23
2023	Q8C2E6	Putative uncharacterized protein OS=Mus	22	23
1247	Q3UAD6	Endoplasmin OS=Mus musculus GN=Hsp	22	22
1271	Q3UBU0	Putative uncharacterized protein OS=Mus	22	22
1627	Q60605-2	Isoform Smooth muscle of Myosin light p	22	22
1789	Q6PHN9	Ras-related protein Rab-35 OS=Mus musc	22	22
2171	Q91V38	Heat shock protein 90, beta (Grp94), mem	22	22
2462	Q9JL26	Formin-like protein 1 OS=Mus musculus	22	22
73	A8IP69	14-3-3 protein gamma OS=Mus musculus	22	21
1458	Q497E9	40S ribosomal protein S8 OS=Mus muscu	22	21
1499	Q540M6	Tumor necrosis factor receptor superfamil	22	17
1615	Q5SXG3	Misshapen-like kinase 1 OS=Mus muscul	22	17
2042	Q8C4K3	Putative uncharacterized protein OS=Mus	22	17
2306	Q9CWJ9	Bifunctional purine biosynthesis protein P	22	17
767	P63094-2	Isoform Gnas-2 of Guanine nucleotide-bi	22	16
657	P47911	60S ribosomal protein L6 OS=Mus muscu	22	15
1617	Q5SZA3	Histone H1.2 OS=Mus musculus GN=His	22	15
1814	Q76MZ3	Serine/threonine-protein phosphatase 2A	22	14
1964	Q8BT07	Centrosomal protein of 55 kDa OS=Mus r	22	14
2022	Q8C2E1	Putative uncharacterized protein OS=Mus	22	14
108	B2RQ80	Tnik protein OS=Mus musculus GN=Tnik	22	13
155	B9EKN8	TRAF2 and NCK interacting kinase OS=M	22	13
1530	Q548T0	Syntaxin binding protein 2, isoform CRA_	22	10
896	Q3TAI8	Putative uncharacterized protein OS=Mus	21	25
1675	Q61696	Heat shock 70 kDa protein 1A OS=Mus m	21	25
1819	Q790I0	Valine--tRNA ligase OS=Mus musculus G	21	24
1861	Q80UE5	Epb4.1l2 protein OS=Mus musculus GN=	21	23
629	P39688-2	Isoform 2 of Tyrosine-protein kinase Fyn	21	20
2386	Q9D8N0	Elongation factor 1-gamma OS=Mus mus	21	20
1557	Q5DQJ3	Capping protein (Actin filament) muscle Z	21	19
771	P63242	Eukaryotic translation initiation factor 5A	21	18
802	P84078	ADP-ribosylation factor 1 OS=Mus muscu	21	18
1155	Q3U344	ADP-ribosylation factor 3 OS=Mus muscu	21	18
725	P62141	Serine/threonine-protein phosphatase PP1	21	17
2220	Q922D8	C-1-tetrahydrofolate synthase, cytoplasmi	21	17

561	P23116	Eukaryotic translation initiation factor 3 s	21	15
1101	Q3TW28	Putative uncharacterized protein OS=Mus	21	15
559	P21956	Lactadherin OS=Mus musculus GN=Mfg	21	14
829	Q01705	Neurogenic locus notch homolog protein	21	14
980	Q3TI61	Putative uncharacterized protein OS=Mus	21	13
2154	Q8VDM4	26S proteasome non-ATPase regulatory s	21	13
976	Q3TI05	Chaperonin containing Tcp1, subunit 6a (	20	25
999	Q3TIX8	Putative uncharacterized protein OS=Mus	20	25
1493	Q52KG9	Chaperonin containing Tcp1, subunit 6a (	20	25
229	E9Q3Q6	CD166 antigen OS=Mus musculus GN=A	20	22
210	E9PWT4	H-2 class I histocompatibility antigen, Q7	20	19
756	P62984	Ubiquitin-60S ribosomal protein L40 OS=	20	19
1601	Q5SQB7	MCG68069 OS=Mus musculus GN=Npm	20	19
2267	Q99PT1	Rho GDP-dissociation inhibitor 1 OS=Mu	20	17
1744	Q6IRU2	Tropomyosin alpha-4 chain OS=Mus mus	20	16
701	P60335	Poly(rC)-binding protein 1 OS=Mus musc	20	14
8	A1L0U3	Histone H3 (Fragment) OS=Mus musculu	20	13
553	P19973	Lymphocyte-specific protein 1 OS=Mus n	20	13
1929	Q8BKC5	Importin-5 OS=Mus musculus GN=Ipo5 I	20	13
100	B1B506	L-selectin OS=Mus musculus GN=Sell PI	20	10
101	B1B507	L-selectin OS=Mus musculus GN=Sell PI	20	10
669	P49710	Hematopoietic lineage cell-specific protei	20	10
2529	Q9WV91	Prostaglandin F2 receptor negative regula	20	7
2193	Q91XV3	Brain acid soluble protein 1 OS=Mus mus	19	31
511	P13597	Intercellular adhesion molecule 1 OS=Mu	19	23
901	Q3TB10	Putative uncharacterized protein (Fragme	19	23
962	Q3THH5	Putative uncharacterized protein OS=Mus	19	23
1203	Q3U7Q9	Putative uncharacterized protein OS=Mus	19	23
1291	Q3UDJ5	Putative uncharacterized protein OS=Mus	19	23
1469	Q4FK06	Icam1 protein OS=Mus musculus GN=Ica	19	23
709	P61161	Actin-related protein 2 OS=Mus musculus	19	19
859	Q0PD50	RAB8A, member RAS oncogene family,	19	18
949	Q3TGU7	Proliferation-associated 2G4 OS=Mus mu	19	18
1134	Q3U0D7	ADP-ribosylation factor 6 OS=Mus musc	19	18
1298	Q3UDZ1	Ras homolog gene family, member G OS=	19	18
776	P68040	Guanine nucleotide-binding protein subun	19	17
1551	Q5BL09	Npm1 protein OS=Mus musculus GN=Np	19	17
2471	Q9QUJ7	Long-chain-fatty-acid--CoA ligase 4 OS=	19	17
476	P07742	Ribonucleoside-diphosphate reductase lar	19	16
524	P14733	Lamin-B1 OS=Mus musculus GN=Lmnb	19	15

558	P21550	Beta-enolase OS=Mus musculus GN=Enc	19	15
643	P43274	Histone H1.4 OS=Mus musculus GN=His	19	14
700	P60122	RuvB-like 1 OS=Mus musculus GN=Ruv	19	14
1337	Q3UJN2	Putative uncharacterized protein OS=Mus	19	14
1670	Q61599	Rho GDP-dissociation inhibitor 2 OS=Mu	19	14
1331	Q3UIT9	Proteasome subunit alpha type OS=Mus n	19	13
2558	Q9Z2U0	Proteasome subunit alpha type-7 OS=Mus	19	13
594	P28667	MARCKS-related protein OS=Mus muscu	19	11
1429	Q3UXQ6	40S ribosomal protein S4 OS=Mus muscu	19	10
1523	Q545F8	40S ribosomal protein S4 OS=Mus muscu	19	10
1528	Q545X8	40S ribosomal protein S4 OS=Mus muscu	19	10
2547	Q9Z179	SHC SH2 domain-binding protein 1 OS=M	19	7
343	G5E829	Plasma membrane calcium-transporting A	18	25
979	Q3TI59	Putative uncharacterized protein OS=Mus	18	24
223	E9Q0U7	Heat shock protein 105 kDa OS=Mus mus	18	23
347	G5E8R2	Tropomyosin 1, alpha, isoform CRA_k OS	18	20
1163	Q3U4D1	Adenosylhomocysteinase OS=Mus muscu	18	19
631	P40142	Transketolase OS=Mus musculus GN=Tk	18	17
1693	Q62261	Spectrin beta chain, non-erythrocytic 1 OS	18	17
484	P09055	Integrin beta-1 OS=Mus musculus GN=Itg	18	16
858	Q0PD49	RAB8B, member RAS oncogene family C	18	16
2214	Q921M7	Protein FAM49B OS=Mus musculus GN=	18	16
1644	Q60864	Stress-induced-phosphoprotein 1 OS=Mus	18	15
1637	Q60737	Casein kinase II subunit alpha OS=Mus m	18	14
1660	Q61177	Casein kinase II alpha subunit OS=Mus m	18	14
2544	Q9Z0P5	Twinfilin-2 OS=Mus musculus GN=Twf2	18	14
2141	Q8R366	Immunoglobulin superfamily member 8 C	18	13
1859	Q80U72	Protein scribble homolog OS=Mus muscu	18	10
565	P24161	T-cell surface glycoprotein CD3 zeta chai	18	9
322	G3UY19	Protein regulator of cytokinesis 1 OS=Mu	18	7
1153	Q3U2W2	MYB binding protein (P160) 1a, isoform (	18	7
1639	Q60767	Lymphocyte antigen 75 OS=Mus musculu	17	26
2076	Q8CE30	Putative uncharacterized protein OS=Mus	17	18
809	P97351	40S ribosomal protein S3a OS=Mus musc	17	17
1176	Q3U5P8	40S ribosomal protein S3a OS=Mus musc	17	17
1740	Q6GT24	Peroxiredoxin 6 OS=Mus musculus GN=I	17	16
1529	Q546H1	Linker for activation of T cells, isoform C	17	15
2020	Q8C2A3	Putative uncharacterized protein OS=Mus	17	15
518	P14148	60S ribosomal protein L7 OS=Mus muscu	17	14
1001	Q3TJ38	Putative uncharacterized protein OS=Mus	17	14

1267	Q3UBI6	Putative uncharacterized protein OS=Mus	17	14
31	A2AF47	Dedicator of cytokinesis protein 11 OS=M	17	13
2290	Q9CQV8	14-3-3 protein beta/alpha OS=Mus muscu	17	13
803	P84084	ADP-ribosylation factor 5 OS=Mus muscu	17	12
1958	Q8BQ30	Phostensin OS=Mus musculus GN=Ppp1r	17	12
2246	Q99KN1	Arrestin domain-containing protein 1 OS=	17	12
1023	Q3TKM9	Actin-related protein 2/3 complex subunit	17	10
2273	Q9CPW4	Actin-related protein 2/3 complex subunit	17	10
1850	Q7TSZ3	Leucyl-tRNA synthetase OS=Mus muscul	17	9
1875	Q80X90	Filamin-B OS=Mus musculus GN=Flnb P	17	3
456	P01590	Interleukin-2 receptor subunit alpha OS=N	16	22
1804	Q6ZWV3	60S ribosomal protein L10 OS=Mus musc	16	19
655	P47757-2	Isoform 2 of F-actin-capping protein subu	16	18
571	P25444	40S ribosomal protein S2 OS=Mus muscu	16	15
825	P99027	60S acidic ribosomal protein P2 OS=Mus	16	15
1028	Q3TL20	Putative uncharacterized protein OS=Mus	16	15
1035	Q3TLE5	Putative uncharacterized protein OS=Mus	16	15
1119	Q3TXS9	Putative uncharacterized protein OS=Mus	16	15
2331	Q9CYL5	Golgi-associated plant pathogenesis-relate	16	15
545	P18181	CD48 antigen OS=Mus musculus GN=Cd	16	13
1771	Q6P905	CD48 antigen OS=Mus musculus GN=Cd	16	13
107	B2M1R7	Poly(RC) binding protein 2 OS=Mus mus	16	12
833	Q02242	Programmed cell death protein 1 OS=Mus	16	12
1688	Q61990	Poly(rC)-binding protein 2 OS=Mus musc	16	12
1689	Q61990-3	Isoform 3 of Poly(rC)-binding protein 2 O	16	12
891	Q3TA56	P-selectin glycoprotein ligand 1 OS=Mus	16	11
1136	Q3U0F1	Putative uncharacterized protein OS=Mus	16	11
1692	Q62170	P-selectin glycoprotein ligand 1 OS=Mus	16	11
2250	Q99L34	Selectin, platelet (P-selectin) ligand OS=M	16	11
2338	Q9CZD3	Glycine--tRNA ligase OS=Mus musculus	16	10
367	J3QNK8	Abl interactor 1 OS=Mus musculus GN=A	16	9
560	P22646	T-cell surface glycoprotein CD3 epsilon c	16	8
2098	Q8CIJ3	Eukaryotic translation initiation factor 3 su	16	6
2101	Q8JZQ9	Eukaryotic translation initiation factor 3 su	16	6
790	P70429	Ena/VASP-like protein OS=Mus musculu	16	3
703	P60766	Cell division control protein 42 homolog (	15	19
2133	Q8R0J7	Vacuolar protein sorting-associated protei	15	17
2135	Q8R1B4	Eukaryotic translation initiation factor 3 su	15	16
1981	Q8BU31	Ras-related protein Rap-2c OS=Mus musc	15	14
420	O54901	OX-2 membrane glycoprotein OS=Mus m	15	13

1495	Q52L97	Importin subunit alpha OS=Mus musculus	15	13
2009	Q8C147	Dedicator of cytokinesis protein 8 OS=Mu	15	13
587	P27773	Protein disulfide-isomerase A3 OS=Mus r	15	12
1234	Q3U9J9	Putative uncharacterized protein OS=Mus	15	12
1505	Q542J3	Protein tyrosine phosphatase receptor type	15	12
321	G3UXZ5	Proteasome activator complex subunit 1 (l	15	11
812	P97371	Proteasome activator complex subunit 1 C	15	11
1732	Q6A0D4	Raftlin OS=Mus musculus GN=Rftn1 PE=	15	11
259	E9QAZ2	Ribosomal protein L15 OS=Mus musculu	15	10
916	Q3TDA7	Protein kinase C and casein kinase substra	15	10
1573	Q5M8Q0	Ribosomal protein L15 OS=Mus musculu	15	10
653	P47741	Tumor necrosis factor receptor superfamil	15	9
663	P48036	Annexin A5 OS=Mus musculus GN=Anx	15	9
1806	Q6ZWX2	Thymosin, beta 4, X chromosome OS=Mu	15	6
1517	Q544H9	Protein phosphatase 1 regulatory inhibitor	15	2
592	P28656	Nucleosome assembly protein 1-like 1 OS	14	19
932	Q3TF41	Nucleosome assembly protein 1-like 1, iso	14	19
1962	Q8BSH9	Nucleosome assembly protein 1-like 1, iso	14	19
722	P62071	Ras-related protein R-Ras2 OS=Mus musc	14	16
586	P27661	Histone H2AX OS=Mus musculus GN=H	14	14
837	Q05144	Ras-related C3 botulinum toxin substrate 2	14	14
945	Q3TGC5	Putative uncharacterized protein OS=Mus	14	12
1442	Q3V117	ATP-citrate synthase OS=Mus musculus C	14	12
1564	Q5EBQ2	MCG7941, isoform CRA_f OS=Mus mus	14	12
1718	Q64735	Complement component receptor 1-like pr	14	12
607	P32883-2	Isoform 2B of GTPase KRas OS=Mus mu	14	11
2013	Q8C1B7	Septin-11 OS=Mus musculus GN=Sept11	14	11
1661	Q61187	Tumor susceptibility gene 101 protein OS	14	10
263	E9QKK8	Phospholipid-transporting ATPase OS=M	14	8
793	P70460	Vasodilator-stimulated phosphoprotein OS	14	8
2493	Q9QZW0	Phospholipid-transporting ATPase 11C O	14	8
651	P46471	26S protease regulatory subunit 7 OS=Mu	14	7
1980	Q8BU30	Isoleucine--tRNA ligase, cytoplasmic OS=	14	7
337	G3X9V0	MCG22048, isoform CRA_a OS=Mus mu	14	6
714	P61226	Ras-related protein Rap-2b OS=Mus musc	13	15
2203	Q91Z67	SLIT-ROBO Rho GTPase-activating prot	13	15
339	G3XA10	Heterogeneous nuclear ribonucleoprotein	13	14
1096	Q3TVV6	Putative uncharacterized protein OS=Mus	13	14
1121	Q3TXW2	Putative uncharacterized protein OS=Mus	13	14
2018	Q8C290	Putative uncharacterized protein OS=Mus	13	14

2162	Q8VEK3	Heterogeneous nuclear ribonucleoprotein	13	14
127	B6ZHC8	Erythrocyte protein band 4.1 OS=Mus mu	13	13
661	P47963	60S ribosomal protein L13 OS=Mus musc	13	13
762	P63028	Translationally-controlled tumor protein C	13	13
1224	Q3U8Y7	Putative uncharacterized protein OS=Mus	13	13
1801	Q6ZWN5	40S ribosomal protein S9 OS=Mus muscu	13	13
1855	Q80T06	Elongation factor 1-delta OS=Mus muscu	13	13
2048	Q8C6A3	Putative uncharacterized protein OS=Mus	13	13
611	P34022	Ran-specific GTPase-activating protein O	13	12
1375	Q3ULZ3	Phosphoserine aminotransferase OS=Mus	13	12
1512	Q543K5	Phosphoserine aminotransferase OS=Mus	13	12
2560	Q9Z2X1	Heterogeneous nuclear ribonucleoprotein	13	12
1160	Q3U3T6	Putative uncharacterized protein OS=Mus	13	11
1282	Q3UD67	Putative uncharacterized protein OS=Mus	13	11
1793	Q6XMP4	Alanyl-tRNA synthase OS=Mus musculus	13	11
1906	Q8BGQ7	Alanine--tRNA ligase, cytoplasmic OS=M	13	11
2244	Q99KH8	Serine/threonine-protein kinase 24 OS=M	13	11
199	E9PUF7	Rho guanine nucleotide exchange factor 1	13	10
527	P15532	Nucleoside diphosphate kinase A OS=Mu	13	10
758	P63001	Ras-related C3 botulinum toxin substrate	13	10
855	Q09143	High affinity cationic amino acid transpor	13	10
1037	Q3TLP8	RAS-related C3 botulinum substrate 1, isc	13	10
1296	Q3UDV5	Putative uncharacterized protein OS=Mus	13	10
1408	Q3UTL4	Putative uncharacterized protein OS=Mus	13	10
1470	Q4FK16	Plscr1 protein OS=Mus musculus GN=Pls	13	10
1567	Q5FWB6	60S acidic ribosomal protein P0 OS=Mus	13	10
1574	Q5M8R8	60S acidic ribosomal protein P0 OS=Mus	13	10
1952	Q8BPG5	Putative uncharacterized protein OS=Mus	13	10
2259	Q99M50	Plscr1 protein OS=Mus musculus GN=Pls	13	10
2383	Q9D859	Putative uncharacterized protein OS=Mus	13	10
2448	Q9JJ00	Phospholipid scramblase 1 OS=Mus musc	13	10
407	O35598	Disintegrin and metalloproteinase domain	13	9
453	P00493	Hypoxanthine-guanine phosphoribosyltra	13	9
572	P25799	Nuclear factor NF-kappa-B p105 subunit	13	9
699	P59999	Actin-related protein 2/3 complex subunit	13	9
911	Q3TCZ2	Solute carrier family 29 (Nucleoside trans	13	9
2007	Q8C129	Leucyl-cystinyl aminopeptidase OS=Mus	13	9
2192	Q91XT3	Stathmin OS=Mus musculus GN=Stmn1	13	9
2243	Q99KF5	Hypoxanthine guanine phosphoribosyl tra	13	9
2369	Q9D2V7	Coronin-7 OS=Mus musculus GN=Coro7	13	9

2370	Q9D3C4	Actin-related protein 2/3 complex subunit	13	9
660	P47962	60S ribosomal protein L5 OS=Mus muscu	13	8
1209	Q3U850	Putative uncharacterized protein OS=Mus	13	8
1448	Q3V3E1	Ubiquitin-associated and SH3 domain-cor	13	7
610	P33174	Chromosome-associated kinesin KIF4 OS	13	6
2446	Q9JIK5	Nucleolar RNA helicase 2 OS=Mus musc	13	6
1900	Q8BG67	Protein EFR3 homolog A OS=Mus muscu	13	3
246	E9Q6R7	Protein Utrn OS=Mus musculus GN=Utrn	13	2
1082	Q3TTA7	E3 ubiquitin-protein ligase CBL-B OS=M	13	0
249	E9Q828	Calcium-transporting ATPase OS=Mus m	12	33
350	H2BL43	Calcium-transporting ATPase OS=Mus m	12	33
103	B2CSK2	Heat shock protein 1-like protein OS=Mu	12	14
162	D3YTN4	GTPase IMAP family member 4 OS=Mus	12	14
533	P16627	Heat shock 70 kDa protein 1-like OS=Mu	12	14
599	P30416	Peptidyl-prolyl cis-trans isomerase FKBP	12	14
1677	Q61735-2	Isoform 2 of Leukocyte surface antigen C	12	14
2496	Q9R0G6	Cartilage oligomeric matrix protein OS=M	12	14
104	B2CY77	Laminin receptor (Fragment) OS=Mus mu	12	13
621	P35979	60S ribosomal protein L12 OS=Mus musc	12	13
678	P51150	Ras-related protein Rab-7a OS=Mus musc	12	12
1044	Q3TMA0	Putative uncharacterized protein OS=Mus	12	12
1328	Q3UII2	Tetraspanin OS=Mus musculus GN=Cd82	12	12
1613	Q5SW88	Protein Rab1a OS=Mus musculus GN=Ra	12	12
1909	Q8BH43	Wiskott-Aldrich syndrome protein family	12	12
2033	Q8C2Q7	Heterogeneous nuclear ribonucleoprotein	12	12
1626	Q5YLW3	40S ribosomal protein S3 OS=Mus muscu	12	11
2027	Q8C2K1	Differentially expressed in FDCP 6 OS=M	12	11
2295	Q9CR16	Peptidyl-prolyl cis-trans isomerase D OS=	12	11
857	Q0PD35	RAB21, member RAS oncogene family O	12	10
1303	Q3UEI6	Putative uncharacterized protein OS=Mus	12	10
1335	Q3UJK2	Putative uncharacterized protein OS=Mus	12	10
1380	Q3UMP4	Plasminogen activator inhibitor 1 RNA-bi	12	10
1409	Q3UTP8	Amino acid transporter OS=Mus musculu	12	10
1998	Q8BXT5	Amino acid transporter OS=Mus musculu	12	10
2326	Q9CY58	Plasminogen activator inhibitor 1 RNA-bi	12	10
2327	Q9CY58-2	Isoform 2 of Plasminogen activator inhibi	12	10
392	O09044	Synaptosomal-associated protein 23 OS=M	12	9
838	Q05816	Fatty acid-binding protein, epidermal OS=	12	9
1190	Q3U6F6	Putative uncharacterized protein OS=Mus	12	9
1204	Q3U7R1	Extended synaptotagmin-1 OS=Mus musc	12	9

1481	Q4VA10	Ras homolog gene family, member f OS=]	12	9
2284	Q9CQI6	Coactosin-like protein OS=Mus musculus	12	9
2362	Q9D1G1	Ras-related protein Rab-1B OS=Mus mus	12	9
2373	Q9D3L3	Synaptosomal-associated protein OS=Mus	12	9
297	F6YVP7	Protein Gm10260 OS=Mus musculus GN=	12	8
1533	Q561N5	40S ribosomal protein S18 OS=Mus musc	12	8
1753	Q6NZJ6	Eukaryotic translation initiation factor 4 g	12	7
394	O09126	Semaphorin-4D OS=Mus musculus GN=S	12	4
1920	Q8BJC1	Putative uncharacterized protein (Fragmer	12	4
2158	Q8VDZ4	Palmitoyltransferase ZDHHC5 OS=Mus r	12	4
555	P20152	Vimentin OS=Mus musculus GN=Vim PE	11	23
601	P31041	T-cell-specific surface glycoprotein CD28	11	16
2072	Q8CDB3	Putative uncharacterized protein OS=Mus	11	16
1440	Q3V014	H-2 class I histocompatibility antigen, D-:	11	15
1046	Q3TMC5	Putative uncharacterized protein OS=Mus	11	14
2441	Q9JHU4	Cytoplasmic dynein 1 heavy chain 1 OS=]	11	13
113	B2RTL6	Thrombospondin 4 OS=Mus musculus GN	11	12
506	P12382	ATP-dependent 6-phosphofructokinase, li	11	12
1251	Q3UAG2	6-phosphogluconate dehydrogenase, decar	11	12
2169	Q91V28	6-phosphogluconate dehydrogenase, decar	11	12
2274	Q9CQ43	Deoxyuridine triphosphatase OS=Mus mu	11	12
2409	Q9DCD0	6-phosphogluconate dehydrogenase, decar	11	12
507	P12815	Programmed cell death protein 6 OS=Mus	11	11
774	P63330	Serine/threonine-protein phosphatase 2A c	11	11
2463	Q9JLB0-2	Isoform Alpha of MAGUK p55 subfamily	11	11
741	P62827	GTP-binding nuclear protein Ran OS=Mu	11	10
763	P63037	DnaJ homolog subfamily A member 1 OS	11	10
792	P70452	Syntaxin-4 OS=Mus musculus GN=Stx4 l	11	10
1374	Q3ULW0	Putative uncharacterized protein OS=Mus	11	10
2506	Q9R1P0	Proteasome subunit alpha type-4 OS=Mus	11	10
29	A2AD84	Serine/threonine-protein kinase 26 OS=M	11	9
30	A2AD85	Serine/threonine-protein kinase 26 OS=M	11	9
2232	Q99020	Heterogeneous nuclear ribonucleoprotein	11	9
2356	Q9D0I9	Arginine--tRNA ligase, cytoplasmic OS=l	11	9
274	E9QMX7	Rho GTPase-activating protein 30 OS=Mu	11	8
828	Q01320	DNA topoisomerase 2-alpha OS=Mus mu	11	8
1170	Q3U4Y3	CD3 antigen gamma polypeptide OS=Mus	11	8
1706	Q640N3	Rho GTPase-activating protein 30 OS=Mu	11	8
1242	Q3UA23	Protein disulfide-isomerase OS=Mus mus	11	7
764	P63038	60 kDa heat shock protein, mitochondrial	11	6

898	Q3TAS3	Stx11 protein OS=Mus musculus GN=Stx	11	6
1184	Q3U5V8	MCG49559 OS=Mus musculus GN=Stx1	11	6
2371	Q9D3G5	Syntaxin-11 OS=Mus musculus GN=Stx1	11	6
2536	Q9Z0F4	Calcium and integrin-binding protein 1 O	11	5
2484	Q9QYI3	DnaJ homolog subfamily C member 7 OS	11	4
1466	Q4FJX4	Csrp1 protein OS=Mus musculus GN=Cs	11	3
1566	Q5F283	Phospholipid scramblase 3 OS=Mus musc	10	13
480	P08228	Superoxide dismutase [Cu-Zn] OS=Mus r	10	12
2455	Q9JKB3	Y-box-binding protein 3 OS=Mus muscul	10	12
860	Q0PD65	RAB2, member RAS oncogene family OS	10	11
1491	Q50HX4	RAB14 protein OS=Mus musculus GN=R	10	11
1799	Q6ZQF2	MKIAA0253 protein (Fragment) OS=Mu	10	11
143	B7ZP22	Heterogeneous nuclear ribonucleoprotein	10	10
442	O88569	Heterogeneous nuclear ribonucleoproteins	10	10
619	P35762	CD81 antigen OS=Mus musculus GN=Cd	10	10
671	P49722	Proteasome subunit alpha type-2 OS=Mus	10	10
1423	Q3UWG5	Tetraspanin OS=Mus musculus GN=Cd81	10	10
1426	Q3UWT6	Proteasome subunit alpha type OS=Mus n	10	10
2361	Q9D1C8	Vacuolar protein sorting-associated protei	10	10
490	P0C0S6	Histone H2A.Z OS=Mus musculus GN=H	10	9
542	P17918	Proliferating cell nuclear antigen OS=Mus	10	9
1594	Q5RJV5	Polypyrimidine tract binding protein 1 OS	10	9
1946	Q8BP47	Asparagine--tRNA ligase, cytoplasmic OS	10	9
2204	Q91ZH2	Proliferating cell nuclear antigen OS=Mus	10	9
306	F8VQC9	Anion exchange protein OS=Mus muscul	10	8
638	P42227	Signal transducer and activator of transcri	10	8
1196	Q3U6S9	Signal transducer and activator of transcri	10	8
1419	Q3UVN5	Putative uncharacterized protein OS=Mus	10	8
1826	Q7TMK9-2	Isoform 2 of Heterogeneous nuclear ribon	10	8
2336	Q9CZ44	NSFL1 cofactor p47 OS=Mus musculus C	10	8
647	P45376	Aldose reductase OS=Mus musculus GN=	10	7
648	P46061	Ran GTPase-activating protein 1 OS=Mus	10	7
681	P53996	Cellular nucleic acid-binding protein OS=	10	7
909	Q3TCL2	Putative uncharacterized protein (Fragmen	10	7
1002	Q3TJ39	RAB5C, member RAS oncogene family, i	10	7
1182	Q3U5V2	Putative uncharacterized protein OS=Mus	10	7
1619	Q5U415	Aldo-keto reductase family 1, member B3	10	7
1795	Q6ZPH4	MKIAA1835 protein (Fragment) OS=Mu	10	7
2200	Q91YS2	Rangap1 protein OS=Mus musculus GN=	10	7
695	P58044	Isopentenyl-diphosphate Delta-isomerase	10	6

850	Q08093	Calponin-2 OS=Mus musculus GN=Cnn2	10	6
1127	Q3TYV5	2',3'-cyclic-nucleotide 3'-phosphodiesteras	10	6
82	B1ASP2	Tyrosine-protein kinase OS=Mus musculu	10	5
94	B1AYC9	Amyloid beta (A4) protein-binding, family	10	5
636	P42209	Septin-1 OS=Mus musculus GN=Sept1 Pl	10	5
1401	Q3URU8	Tyrosine-protein kinase OS=Mus musculu	10	5
2045	Q8C503	Signaling threshold-regulating transmemb	10	5
97	B1AZ46	Brain-specific angiogenesis inhibitor 1-ass	10	3
1422	Q3UW53	Protein Niban OS=Mus musculus GN=Fa	10	3
1934	Q8BKX1	Brain-specific angiogenesis inhibitor 1-ass	10	3
1935	Q8BKX1-2	Isoform 2 of Brain-specific angiogenesis i	10	3
1936	Q8BKX1-3	Isoform 3 of Brain-specific angiogenesis i	10	3
2429	Q9ES61	Chandra protein OS=Mus musculus GN=I	10	3
540	P17809	Solute carrier family 2, facilitated glucose	9	11
1151	Q3U2J2	Putative uncharacterized protein OS=Mus	9	11
1461	Q4FJL0	RAB10, member RAS oncogene family O	9	11
1550	Q58EW0	60S ribosomal protein L18 OS=Mus musc	9	11
1709	Q642K1	Ribosomal protein L18 OS=Mus musculu	9	11
380	O08553	Dihydropyrimidinase-related protein 2 OS	9	10
517	P14131	40S ribosomal protein S16 OS=Mus musc	9	10
2311	Q9CWW5	Putative uncharacterized protein OS=Mus	9	10
2324	Q9CXZ9	Putative uncharacterized protein OS=Mus	9	10
146	B7ZWC4	Insulin-like growth factor 2 receptor OS=I	9	9
278	E9QNY6	B- and T-lymphocyte attenuator OS=Mus	9	9
430	O70194	Eukaryotic translation initiation factor 3 su	9	9
713	P61222	ATP-binding cassette sub-family E memb	9	9
734	P62334	26S protease regulatory subunit 10B OS=I	9	9
848	Q07113	Cation-independent mannose-6-phosphate	9	9
997	Q3TIU8	Putative uncharacterized protein OS=Mus	9	9
1750	Q6NXX7	Abce1 protein (Fragment) OS=Mus muscu	9	9
1811	Q6ZWZ6	40S ribosomal protein S12 OS=Mus musc	9	9
1847	Q7TSA3	B- and T-lymphocyte attenuator OS=Mus	9	9
1848	Q7TSA3-3	Isoform 3 of B- and T-lymphocyte attenua	9	9
2468	Q9JM76	Actin-related protein 2/3 complex subunit	9	9
433	O70296	G protein-coupled receptor kinase 6 OS=M	9	8
478	P08030	Adenine phosphoribosyltransferase OS=M	9	8
556	P20334	Tumor necrosis factor receptor superfamil	9	8
1095	Q3TVR4	Putative uncharacterized protein OS=Mus	9	8
1397	Q3UQM7	Putative uncharacterized protein OS=Mus	9	8
2546	Q9Z127	Large neutral amino acids transporter sma	9	8

175	D3Z0N6	Tetraspanin (Fragment) OS=Mus musculu	9	7
227	E9Q2X8	Tetraspanin OS=Mus musculus GN=Cd37	9	7
1147	Q3U1W9	Tetraspanin OS=Mus musculus GN=Cd37	9	7
1161	Q3U429	Tetraspanin OS=Mus musculus GN=Cd37	9	7
1489	Q505B1	Rpl17 protein (Fragment) OS=Mus muscu	9	7
1667	Q61470	Leukocyte antigen CD37 OS=Mus muscu	9	7
1812	Q6ZWZ7	60S ribosomal protein L17 OS=Mus musc	9	7
1868	Q80V08	Rpl17 protein (Fragment) OS=Mus muscu	9	7
2126	Q8QZY1	Eukaryotic translation initiation factor 3 su	9	7
2270	Q9CPR4	60S ribosomal protein L17 OS=Mus musc	9	7
2297	Q9CR57	60S ribosomal protein L14 OS=Mus musc	9	7
2354	Q9D0E1	Heterogeneous nuclear ribonucleoprotein	9	7
378	N0E4C0	Casein kinase II subunit beta OS=Mus mu	9	6
464	P05063	Fructose-bisphosphate aldolase C OS=Mu	9	6
729	P62281	40S ribosomal protein S11 OS=Mus musc	9	6
759	P63005	Platelet-activating factor acetylhydrolase I	9	6
775	P67871	Casein kinase II subunit beta OS=Mus mu	9	6
990	Q3TIK0	Platelet-activating factor acetylhydrolase I	9	6
1009	Q3TJG1	Platelet-activating factor acetylhydrolase I	9	6
1318	Q3UGR6	Platelet-activating factor acetylhydrolase I	9	6
576	P26350	Prothymosin alpha OS=Mus musculus GN	9	5
602	P31230	Aminoacyl tRNA synthase complex-intera	9	5
742	P62830	60S ribosomal protein L23 OS=Mus musc	9	5
1189	Q3U6E4	Putative uncharacterized protein OS=Mus	9	5
1435	Q3UZG4	Aminoacyl tRNA synthase complex-intera	9	5
2470	Q9JMH6	Thioredoxin reductase 1, cytoplasmic OS=	9	5
567	P24527	Leukotriene A-4 hydrolase OS=Mus musc	9	4
595	P28843	Dipeptidyl peptidase 4 OS=Mus musculus	9	4
622	P37217	Early activation antigen CD69 OS=Mus m	9	4
1069	Q3TR43	Putative uncharacterized protein OS=Mus	9	4
1511	Q543H0	MCG16662 OS=Mus musculus GN=Srm	9	4
2513	Q9R1T4	Septin-6 OS=Mus musculus GN=Sept6 Pl	9	4
2535	Q9Z0E6	Guanylate-binding protein 1 OS=Mus mu	9	4
2565	S4R219	DENN domain-containing protein 2D OS=	9	4
827	Q00651	Integrin alpha-4 OS=Mus musculus GN=I	9	3
2170	Q91V35	Receptor-type tyrosine-protein phosphatas	9	3
1794	Q6ZPE2	Myotubularin-related protein 5 OS=Mus m	9	2
418	O54824	Pro-interleukin-16 OS=Mus musculus GN	9	0
1396	Q3UQ44	Ras GTPase-activating-like protein IQGA	8	16
847	Q07076	Annexin A7 OS=Mus musculus GN=Anx	8	11

1004	Q3TJ49	Annexin OS=Mus musculus GN=Anxa7 I	8	11
1427	Q3UWW9	Putative uncharacterized protein OS=Mus	8	11
1898	Q8BG32	26S proteasome non-ATPase regulatory su	8	11
2218	Q922A2	Annexin OS=Mus musculus GN=Anxa7 I	8	11
1895	Q8BFY9	Transportin-1 OS=Mus musculus GN=Tn	8	10
1896	Q8BFY9-2	Isoform 2 of Transportin-1 OS=Mus musc	8	10
1102	Q3TW51	Putative uncharacterized protein OS=Mus	8	9
1436	Q3UZI3	Putative uncharacterized protein OS=Mus	8	9
1816	Q78PY7	Staphylococcal nuclease domain-containi	8	9
2399	Q9DBR7	Protein phosphatase 1 regulatory subunit 1	8	9
289	F6RPJ9	Insulin-degrading enzyme (Fragment) OS	8	8
635	P42208	Septin-2 OS=Mus musculus GN=Sept2 P	8	8
927	Q3TEK8	CD8 antigen, beta chain 1 OS=Mus muscu	8	8
2491	Q9QZQ8	Core histone macro-H2A.1 OS=Mus musc	8	8
2492	Q9QZQ8-2	Isoform 1 of Core histone macro-H2A.1 C	8	8
401	O35379	Multidrug resistance-associated protein 1	8	7
673	P50396	Rab GDP dissociation inhibitor alpha OS=	8	7
939	Q3TFF0	Putative uncharacterized protein OS=Mus	8	7
1059	Q3TNN6	Putative uncharacterized protein OS=Mus	8	7
1561	Q5DW69	CD226 antigen OS=Mus musculus GN=C	8	7
1571	Q5I0T8	Ribosomal protein L19 OS=Mus musculu	8	7
1802	Q6ZWQ9	MCG5400 OS=Mus musculus GN=Myl12	8	7
2252	Q99LB4	Capping protein (Actin filament), gelsolin	8	7
2485	Q9QYJ0	DnaJ homolog subfamily A member 2 OS	8	7
446	O88890	SH2 domain-containing protein 1A OS=M	8	6
521	P14211	Calreticulin OS=Mus musculus GN=Calr	8	6
568	P24547	Inosine-5'-monophosphate dehydrogenase	8	6
991	Q3TIK8	Putative uncharacterized protein OS=Mus	8	6
1236	Q3U9N8	Inosine-5'-monophosphate dehydrogenase	8	6
1286	Q3UDF8	Putative uncharacterized protein OS=Mus	8	6
1329	Q3UIJ2	Putative uncharacterized protein OS=Mus	8	6
1424	Q3UWP8	Putative uncharacterized protein (Fragmen	8	6
1815	Q78HU3	Multivesicular body subunit 12A OS=Mu	8	6
2008	Q8C145	Zinc transporter ZIP6 OS=Mus musculus	8	6
2423	Q9ERK4	Exportin-2 OS=Mus musculus GN=Cse1l	8	6
2543	Q9Z0N1	Eukaryotic translation initiation factor 2 su	8	6
2569	W0BZ77	SLAM-associated protein isoform SAP-2	8	6
856	Q0GUM3	Interferon-gamma-inducible p47 GTPase	8	5
1087	Q3TUI9	Proteasome subunit alpha type OS=Mus n	8	5
1090	Q3TUX3	Proteasome subunit alpha type OS=Mus n	8	5

1135	Q3U0E8	GRB2-related adaptor protein 2 OS=Mus	8	5
1546	Q58E35	60S acidic ribosomal protein P1 OS=Mus	8	5
1614	Q5SWR1	AP complex subunit beta OS=Mus muscu	8	5
1888	Q811D0-2	Isoform 2 of Disks large homolog 1 OS=M	8	5
1889	Q811D0-3	Isoform 3 of Disks large homolog 1 OS=M	8	5
2394	Q9DBG3	AP-2 complex subunit beta OS=Mus musc	8	5
2559	Q9Z2U1	Proteasome subunit alpha type-5 OS=Mus	8	5
588	P28063	Proteasome subunit beta type-8 OS=Mus m	8	4
1327	Q3UIG8	Putative uncharacterized protein OS=Mus	8	4
1404	Q3US32	Putative uncharacterized protein OS=Mus	8	4
1854	Q80SZ7	Guanine nucleotide-binding protein G(I)/G	8	4
2002	Q8BYC6	Serine/threonine-protein kinase TAO3 OS	8	4
87	B1ATC0	Transmembrane channel-like protein OS=	8	3
1364	Q3ULG4	Putative uncharacterized protein OS=Mus	8	3
1730	Q6A0A3	MKIAA0209 protein (Fragment) OS=Mus	8	3
1809	Q6ZWY8	Thymosin beta-10 OS=Mus musculus GN	8	3
2036	Q8C3J5	Dedicator of cytokinesis protein 2 OS=Mu	8	3
519	P14152	Malate dehydrogenase, cytoplasmic OS=M	8	2
805	P86176	T-cell immunoreceptor with Ig and ITIM d	8	0
824	P99026	Proteasome subunit beta type-4 OS=Mus m	8	0
1974	Q8BTU5	Proteasome subunit alpha type OS=Mus m	8	0
2509	Q9R1P4	Proteasome subunit alpha type-1 OS=Mus	8	0
95	B1AZ14	Cordon-bleu protein-like 1 OS=Mus musc	8	0
96	B1AZ15	Cordon-bleu protein-like 1 OS=Mus musc	8	0
1428	Q3UXP2	Putative uncharacterized protein OS=Mus	7	11
1593	Q5RJV4	Phosphoglucomutase 2 OS=Mus musculu	7	11
1720	Q66JR7	Pgm2 protein (Fragment) OS=Mus muscu	7	11
1837	Q7TNU0	Pgm2 protein (Fragment) OS=Mus muscu	7	11
2355	Q9D0F9	Phosphoglucomutase-1 OS=Mus musculu	7	11
1243	Q3UA53	Putative uncharacterized protein OS=Mus	7	10
1471	Q4FK49	Inorganic pyrophosphatase OS=Mus musc	7	10
2000	Q8BY89	Choline transporter-like protein 2 OS=Mu	7	10
431	O70251	Elongation factor 1-beta OS=Mus muscul	7	9
645	P43276	Histone H1.5 OS=Mus musculus GN=His	7	9
773	P63321	Ras-related protein Ral-A OS=Mus muscu	7	9
874	Q1WWK3	Hist1h1b protein (Fragment) OS=Mus mu	7	9
2323	Q9CXY0	Putative uncharacterized protein OS=Mus	7	9
183	D3Z5I1	Zinc finger CCCH-type antiviral protein 1	7	8
233	E9Q414	Apolipoprotein B-100 OS=Mus musculus	7	8
715	P61255	60S ribosomal protein L26 OS=Mus musc	7	8

1118	Q3TXS7	26S proteasome non-ATPase regulatory sı	7	8
260	E9QB02	Methionine--tRNA ligase, cytoplasmic OS	7	7
696	P58069	Ras GTPase-activating protein 2 OS=Mus	7	7
747	P62855	40S ribosomal protein S26 OS=Mus musc	7	7
753	P62918	60S ribosomal protein L8 OS=Mus muscu	7	7
813	P97384	Annexin A11 OS=Mus musculus GN=An	7	7
818	P97822	Acidic leucine-rich nuclear phosphoprotei	7	7
1110	Q3TX26	Putative uncharacterized protein OS=Mus	7	7
1504	Q542I9	26S protease regulatory subunit 4 OS=Mu	7	7
1565	Q5EBQ6	60S ribosomal protein L9 OS=Mus muscu	7	7
1580	Q5M9L7	40S ribosomal protein S17 OS=Mus musc	7	7
1655	Q61081	Hsp90 co-chaperone Cdc37 OS=Mus mus	7	7
1724	Q68FL6	Methionine--tRNA ligase, cytoplasmic OS	7	7
1968	Q8BT90	Putative uncharacterized protein (Fragmer	7	7
2099	Q8CIN4	Serine/threonine-protein kinase PAK 2 OS	7	7
282	F5BFH0	C-type lectin domain family 2 member D	7	6
463	P04370	Myelin basic protein OS=Mus musculus G	7	6
633	P41105	60S ribosomal protein L28 OS=Mus musc	7	6
751	P62889	60S ribosomal protein L30 OS=Mus musc	7	6
846	Q06138	Calcium-binding protein 39 OS=Mus mus	7	6
1003	Q3TJ43	Putative uncharacterized protein OS=Mus	7	6
1025	Q3TKU6	Putative uncharacterized protein OS=Mus	7	6
1071	Q3TRJ1	Vacuolar protein sorting 35, isoform CRA	7	6
1554	Q5D096	Myelin basic protein OS=Mus musculus G	7	6
1584	Q5M9N5	Ribosomal protein L28 OS=Mus musculu	7	6
2160	Q8VE70	Programmed cell death protein 10 OS=Mu	7	6
2472	Q9QUM4-2	Isoform Short of Signaling lymphocytic ac	7	6
9	A1L333	DEAD (Asp-Glu-Ala-Asp) box polypeptic	7	5
238	E9Q5A0	60S ribosomal protein L13a OS=Mus mus	7	5
437	O70569	Ribosomal protein S14 OS=Mus musculus	7	5
691	P56399	Ubiquitin carboxyl-terminal hydrolase 5 C	7	5
727	P62264	40S ribosomal protein S14 OS=Mus musc	7	5
1167	Q3U4W8	Ubiquitin carboxyl-terminal hydrolase 5 C	7	5
1441	Q3V0Z8	Putative uncharacterized protein (Fragmer	7	5
1618	Q5U222	Ddx5 protein (Fragment) OS=Mus muscu	7	5
1673	Q61656	Probable ATP-dependent RNA helicase D	7	5
1766	Q6P5F9	Exportin-1 OS=Mus musculus GN=Xpo1	7	5
1972	Q8BTS0	DEAD (Asp-Glu-Ala-Asp) box polypeptic	7	5
2538	Q9Z0H4-4	Isoform 4 of CUGBP Elav-like family me	7	5
2539	Q9Z0H4-5	Isoform 5 of CUGBP Elav-like family me	7	5

2540	Q9Z0H4-7	Isoform 7 of CUGBP Elav-like family me	7	5
55	A2BE93	Protein SET (Fragment) OS=Mus muscul	7	4
86	B1ATB3	Transmembrane channel-like protein OS=	7	4
118	B2RUK2	Erbb2 interacting protein OS=Mus muscu	7	4
499	P11157	Ribonucleoside-diphosphate reductase sub	7	4
580	P26883	Peptidyl-prolyl cis-trans isomerase FKBP	7	4
590	P28474	Alcohol dehydrogenase class-3 OS=Mus	7	4
817	P97814	Proline-serine-threonine phosphatase-inte	7	4
886	Q3T9S3	SET translocation OS=Mus musculus GN	7	4
972	Q3THV8	Putative uncharacterized protein OS=Mus	7	4
2161	Q8VEK0	Cell cycle control protein 50A OS=Mus n	7	4
2360	Q9D1A2	Cytosolic non-specific dipeptidase OS=M	7	4
366	J3QNG0	MCG15755 OS=Mus musculus GN=Gm5	7	3
419	O54890	Integrin beta-3 OS=Mus musculus GN=It	7	3
531	P16460	Argininosuccinate synthase OS=Mus mus	7	3
1141	Q3U1F9	Phosphoprotein associated with glycosphi	7	3
1304	Q3UEJ7	Putative uncharacterized protein OS=Mus	7	3
1484	Q4VA93	Protein kinase C OS=Mus musculus GN=	7	3
1527	Q545V8	Casein kinase II subunit alpha' OS=Mus n	7	3
1624	Q5Y5T1	Probable palmitoyltransferase ZDHHC20	7	3
1625	Q5Y5T1-2	Isoform 2 of Probable palmitoyltransferas	7	3
1776	Q6P9R2	Serine/threonine-protein kinase OSR1 OS	7	3
1796	Q6ZPX7	MKIAA1101 protein (Fragment) OS=Mu	7	3
18	A2A6U3	Septin-9 OS=Mus musculus GN=Sept9 P	7	2
1862	Q80UG5	Septin-9 OS=Mus musculus GN=Sept9 P	7	2
1000	Q3TJ01	tRNA-splicing ligase RtcB homolog OS=	7	0
644	P43275	Histone H1.1 OS=Mus musculus GN=His	7	0
1376	Q3UM23	Putative uncharacterized protein OS=Mus	6	17
881	Q3T9A2	SLAM family member 7 OS=Mus muscul	6	12
1912	Q8BHK6	SLAM family member 7 OS=Mus muscul	6	12
2385	Q9D8B3	Charged multivesicular body protein 4b O	6	10
93	B1AWE0	Clathrin light chain A OS=Mus musculus	6	9
968	Q3THP1	Putative uncharacterized protein OS=Mus	6	9
971	Q3THU7	Putative uncharacterized protein OS=Mus	6	9
1285	Q3UDF3	Putative uncharacterized protein OS=Mus	6	9
1672	Q61655	ATP-dependent RNA helicase DDX19A (	6	9
1751	Q6NXZ0	Dipeptidylpeptidase 3 OS=Mus musculus	6	9
1786	Q6PFA2	Clathrin light chain A OS=Mus musculus	6	9
1979	Q8BU29	Dipeptidylpeptidase 3, isoform CRA_c O	6	9
2245	Q99KK7	Dipeptidyl peptidase 3 OS=Mus musculus	6	9

1552	Q5BLJ9	60S ribosomal protein L27 OS=Mus musc	6	8
1950	Q8BPF4	Putative uncharacterized protein OS=Mus	6	8
2325	Q9CY06	Putative uncharacterized protein OS=Mus	6	8
2476	Q9QWJ3	Alpha-1-globin (Fragment) OS=Mus mus	6	8
15	A2A6A6	Inducible T-cell co-stimulator OS=Mus m	6	7
16	A2A6A7	Inducible T-cell co-stimulator OS=Mus m	6	7
243	E9Q616	Protein Ahnak OS=Mus musculus GN=Al	6	7
447	O88952	Protein lin-7 homolog C OS=Mus muscul	6	7
460	P01887	Beta-2-microglobulin OS=Mus musculus	6	7
466	P05201	Aspartate aminotransferase, cytoplasmic (	6	7
529	P16045	Galectin-1 OS=Mus musculus GN=Lgals:	6	7
770	P63213	Guanine nucleotide-binding protein G(I)/(	6	7
998	Q3TIV6	Lysine--tRNA ligase OS=Mus musculus (	6	7
1020	Q3TK95	Eukaryotic translation initiation factor 4E	6	7
1084	Q3TTY6	Putative uncharacterized protein (Fragmer	6	7
1452	Q3V3X2	Putative uncharacterized protein OS=Mus	6	7
1575	Q5M8R9	Farnesyl diphosphate synthetase OS=Mus	6	7
1609	Q5SUZ7	Inducible T-cell co-stimulator OS=Mus m	6	7
1610	Q5SUZ8	Inducible T-cell costimulator OS=Mus mu	6	7
1853	Q80SW1	Putative adenosylhomocysteinase 2 OS=M	6	7
2015	Q8C1V4	Lysine--tRNA ligase OS=Mus musculus (	6	7
2019	Q8C292	Lysine--tRNA ligase OS=Mus musculus (	6	7
2080	Q8CFE6	Sodium-coupled neutral amino acid transp	6	7
2210	Q920E5	Farnesyl pyrophosphate synthase OS=Mu	6	7
2473	Q9QUM9	Proteasome subunit alpha type-6 OS=Mus	6	7
2488	Q9QZE5	Coatomer subunit gamma-1 OS=Mus mus	6	7
395	O09167	60S ribosomal protein L21 OS=Mus musc	6	6
493	P10639	Thioredoxin OS=Mus musculus GN=Txn	6	6
612	P34884	Macrophage migration inhibitory factor O	6	6
634	P41241	Tyrosine-protein kinase CSK OS=Mus mu	6	6
746	P62852	40S ribosomal protein S25 OS=Mus musc	6	6
1416	Q3UVH2	Tyrosine-protein kinase OS=Mus musculu	6	6
1474	Q4FZE6	40S ribosomal protein S7 OS=Mus muscu	6	6
1641	Q60770	Syntaxin-binding protein 3 OS=Mus musc	6	6
1765	Q6P5F7	Protein tweety homolog 3 OS=Mus muscu	6	6
2212	Q921F2	TAR DNA-binding protein 43 OS=Mus m	6	6
2285	Q9CQM8	60S ribosomal protein L21 OS=Mus musc	6	6
2330	Q9CYG7	Mitochondrial import receptor subunit TO	6	6
2424	Q9ERL7	Glia maturation factor gamma OS=Mus m	6	6
71	A7VJ98	Glia maturation factor beta OS=Mus musc	6	5

172	D3YZ61	Complement C1q tumor necrosis factor-re	6	5
1089	Q3TUN5	Putative uncharacterized protein OS=Mus	6	5
1173	Q3U561	Ribosomal protein OS=Mus musculus GN	6	5
1874	Q80X87	Glia maturation factor, beta OS=Mus mus	6	5
1985	Q8BVF6	Putative uncharacterized protein OS=Mus	6	5
2102	Q8K094	Nectin-2 OS=Mus musculus GN=Pvr PE=	6	5
2108	Q8K1I7	WAS/WASL-interacting protein family m	6	5
2117	Q8K2Y3	Protein eva-1 homolog B OS=Mus muscu	6	5
2128	Q8R010	Aminoacyl tRNA synthase complex-intera	6	5
2186	Q91WP1	Cd155 OS=Mus musculus GN=Pvr PE=2	6	5
2283	Q9CQI3	Glia maturation factor beta OS=Mus musc	6	5
2426	Q9ES30	Complement C1q tumor necrosis factor-re	6	5
501	P11440	Cyclin-dependent kinase 1 OS=Mus musc	6	4
716	P61290	Proteasome activator complex subunit 3 C	6	4
726	P62196	26S protease regulatory subunit 8 OS=Mu	6	4
730	P62301	40S ribosomal protein S13 OS=Mus musc	6	4
786	P70315	Wiskott-Aldrich syndrome protein homolo	6	4
918	Q3TDD8	Putative uncharacterized protein OS=Mus	6	4
935	Q3TFA9	Putative uncharacterized protein OS=Mus	6	4
1078	Q3TSY9	Putative uncharacterized protein OS=Mus	6	4
1108	Q3TWZ3	Putative uncharacterized protein OS=Mus	6	4
1316	Q3UGC0	Putative uncharacterized protein OS=Mus	6	4
1903	Q8BGD9	Eukaryotic translation initiation factor 4B	6	4
2183	Q91W53	Golgin subfamily A member 7 OS=Mus n	6	4
2239	Q99JW7	Cdc2a protein (Fragment) OS=Mus muscu	6	4
2438	Q9JHJ0	Tropomodulin-3 OS=Mus musculus GN=	6	4
2490	Q9QZI9	Serine incorporator 3 OS=Mus musculus (	6	4
2500	Q9R0P5	Destrin OS=Mus musculus GN=Dstn PE=	6	4
1	A0A0G2JG10	Pre-mRNA-splicing factor ATP-dependen	6	3
167	D3YW52	Alpha-2-macroglobulin OS=Mus musculu	6	3
313	F8WIV5	Dynamin-2 OS=Mus musculus GN=Dnm2	6	3
481	P08249	Malate dehydrogenase, mitochondrial OS=	6	3
624	P39054	Dynamin-2 OS=Mus musculus GN=Dnm2	6	3
625	P39054-2	Isoform 2 of Dynamin-2 OS=Mus muscul	6	3
632	P40240	CD9 antigen OS=Mus musculus GN=Cd9	6	3
888	Q3T9X3	Dynamin-2 OS=Mus musculus GN=Dnm2	6	3
937	Q3TFE5	Putative uncharacterized protein OS=Mus	6	3
1040	Q3TLX1	Putative uncharacterized protein OS=Mus	6	3
1297	Q3UDX4	Putative uncharacterized protein (Fragmen	6	3
1343	Q3UJS6	Putative uncharacterized protein OS=Mus	6	3

1354	Q3UKJ6	DEAH (Asp-Glu-Ala-His) box polypeptid	6	3
1459	Q497W9	DEAH (Asp-Glu-Ala-His) box polypeptid	6	3
1659	Q61171	Peroxiredoxin-2 OS=Mus musculus GN=I	6	3
1686	Q61838	Alpha-2-macroglobulin OS=Mus musculu	6	3
2032	Q8C2Q0	Putative uncharacterized protein OS=Mus	6	3
2049	Q8C6F4	Putative uncharacterized protein OS=Mus	6	3
2051	Q8C708	Transmembrane protein C16orf54 homolc	6	3
2059	Q8CAG6	Pleckstrin OS=Mus musculus GN=Plek P	6	3
2199	Q91YR1	Twinfilin-1 OS=Mus musculus GN=Twf1	6	3
2249	Q99KQ4	Nicotinamide phosphoribosyltransferase (	6	3
2280	Q9CQD1	Ras-related protein Rab-5A OS=Mus mus	6	3
2380	Q9D7F5	Putative uncharacterized protein OS=Mus	6	3
2439	Q9JHK5	Pleckstrin OS=Mus musculus GN=Plek P	6	3
202	E9PV41	Protein diaphanous homolog 1 OS=Mus n	6	2
212	E9PXV7	Protein diaphanous homolog 1 OS=Mus n	6	2
387	O08808	Protein diaphanous homolog 1 OS=Mus n	6	2
516	P14115	60S ribosomal protein L27a OS=Mus mus	6	2
893	Q3TA88	Putative uncharacterized protein OS=Mus	6	2
965	Q3THK7	GMP synthase [glutamine-hydrolyzing] O	6	2
1549	Q58EV4	Proteasome subunit alpha type OS=Mus n	6	2
1558	Q5DTQ4	MKIAA4062 protein (Fragment) OS=Mus	6	2
1598	Q5RL55	ATP-binding cassette, sub-family F (GCN	6	2
1760	Q6P542	ATP-binding cassette sub-family F memb	6	2
1774	Q6P9N1	Hyccin OS=Mus musculus GN=Fam126a	6	2
2146	Q8R550	SH3 domain-containing kinase-binding pr	6	2
2410	Q9DCD8	Proteasome subunit alpha type OS=Mus n	6	2
1290	Q3UDI8	DNA helicase OS=Mus musculus GN=M	6	0
1443	Q3V122	DNA helicase OS=Mus musculus GN=M	6	0
1687	Q61881	DNA replication licensing factor MCM7 (	6	0
408	O35601	FYN-binding protein OS=Mus musculus (	6	0
435	O70400	PDZ and LIM domain protein 1 OS=Mus	6	0
390	O08917	Flotillin-1 OS=Mus musculus GN=Flot1 I	5	13
115	B2RUC7	Serine-threonine kinase receptor-associate	5	11
674	P50431	Serine hydroxymethyltransferase, cytosoli	5	10
2068	Q8CCG5	Putative uncharacterized protein OS=Mus	5	10
2309	Q9CWR5	Serine hydroxymethyltransferase OS=Mu	5	10
2447	Q9JIW9	Ras-related protein Ral-B OS=Mus muscu	5	10
735	P62482	Voltage-gated potassium channel subunit	5	8
815	P97429	Annexin A4 OS=Mus musculus GN=Anx	5	8
1395	Q3UPV6	Voltage-gated potassium channel subunit	5	8

1829	Q7TMN7	Annexin OS=Mus musculus GN=Anxa4 I	5	8
2172	Q91V55	40S ribosomal protein S5 OS=Mus muscu	5	8
1576	Q5M9K7	40S ribosomal protein S10 OS=Mus musc	5	7
57	A2RS22	Coronin OS=Mus musculus GN=Coro1b I	5	6
530	P16125	L-lactate dehydrogenase B chain OS=Mus	5	6
551	P19157	Glutathione S-transferase P 1 OS=Mus mu	5	6
637	P42225	Signal transducer and activator of transcri	5	6
1317	Q3UGH8	Putative uncharacterized protein (Fragmen	5	6
1525	Q545P4	Tumor necrosis factor receptor superfamil	5	6
1907	Q8BGW0	Protein THEMIS OS=Mus musculus GN=	5	6
570	P25206	DNA replication licensing factor MCM3 (	5	5
783	P70195	Proteasome subunit beta type-7 OS=Mus r	5	5
981	Q3TI69	DNA dC->dU-editing enzyme APOBEC-3	5	5
1219	Q3U8R9	Putative uncharacterized protein OS=Mus	5	5
1363	Q3ULD6	DNA helicase OS=Mus musculus GN=Mc	5	5
1384	Q3UNH3	Putative uncharacterized protein (Fragmen	5	5
1772	Q6P9J9	Anoctamin-6 OS=Mus musculus GN=Anc	5	5
1797	Q6ZQ38	Cullin-associated NEDD8-dissociated pro	5	5
2074	Q8CDN6	Thioredoxin-like protein 1 OS=Mus musc	5	5
2237	Q99JI4	26S proteasome non-ATPase regulatory su	5	5
2241	Q99JX4	Eukaryotic translation initiation factor 3 su	5	5
2392	Q9DB05	Alpha-soluble NSF attachment protein OS	5	5
2419	Q9EPU0	Regulator of nonsense transcripts 1 OS=M	5	5
2518	Q9WTR1	Transient receptor potential cation channe	5	5
88	B1ATD2	Lymphocyte cytosolic protein 2 OS=Mus	5	4
159	C6EQJ5	ASL1/3110003A17Rik fusion protein OS=	5	4
273	E9QMV2	Costars family protein ABRACL OS=Mus	5	4
286	F6QX71	Lck-interacting transmembrane adapter 1	5	4
309	F8WHL2	Coatomer subunit alpha OS=Mus musculu	5	4
393	O09061	Proteasome subunit beta type-1 OS=Mus r	5	4
422	O54988	STE20-like serine/threonine-protein kinas	5	4
523	P14685	26S proteasome non-ATPase regulatory su	5	4
536	P17426	AP-2 complex subunit alpha-1 OS=Mus n	5	4
557	P20934	Protein EVI2A OS=Mus musculus GN=E	5	4
728	P62267	40S ribosomal protein S23 OS=Mus musc	5	4
795	P70670	Nascent polypeptide-associated complex s	5	4
904	Q3TBB7	Putative uncharacterized protein OS=Mus	5	4
995	Q3TIS3	Putative uncharacterized protein OS=Mus	5	4
1126	Q3TYS4	Putative uncharacterized protein OS=Mus	5	4
1352	Q3UKB4	Putative uncharacterized protein OS=Mus	5	4

1370	Q3ULL5	Eif2s2 protein OS=Mus musculus GN=Ei	5	4
1456	Q497E1	Ribosomal protein S23 OS=Mus musculu	5	4
1468	Q4FJZ4	Wars protein OS=Mus musculus GN=Wa	5	4
1642	Q60787	Lymphocyte cytosolic protein 2 OS=Mus	5	4
1679	Q61739-2	Isoform Alpha-6X1A of Integrin alpha-6 (	5	4
1705	Q63ZW9	Copa protein (Fragment) OS=Mus muscul	5	4
1759	Q6P4T2	U5 small nuclear ribonucleoprotein 200 k	5	4
1764	Q6P5F6	Zinc transporter ZIP10 OS=Mus musculu	5	4
1813	Q71FD5	E3 ubiquitin-protein ligase ZNRF2 OS=M	5	4
1926	Q8BK46	Putative uncharacterized protein OS=Mus	5	4
1969	Q8BTF0	Coatomer subunit alpha OS=Mus musculu	5	4
2096	Q8CIE6	Coatomer subunit alpha OS=Mus musculu	5	4
2375	Q9D662	Protein transport protein Sec23B OS=Mus	5	4
2487	Q9QZD9	Eukaryotic translation initiation factor 3 s	5	4
36	A2AI08	Taperin OS=Mus musculus GN=Tprn PE=	5	3
121	B2RWW6	GCN1 general control of amino-acid synt	5	3
122	B2RX66	MCG124812 OS=Mus musculus GN=Tac	5	3
131	B7ZCP4	Copine-1 OS=Mus musculus GN=Cpne1	5	3
203	E9PVA8	Protein Gcn1l1 OS=Mus musculus GN=G	5	3
232	E9Q3X0	Major vault protein OS=Mus musculus G	5	3
269	E9QM38	Solute carrier family 12 member 2 OS=M	5	3
344	G5E866	Splicing factor 3B subunit 1 OS=Mus mu	5	3
974	Q3THX5	Putative uncharacterized protein OS=Mus	5	3
1323	Q3UHQ5	Putative uncharacterized protein OS=Mus	5	3
1359	Q3UL30	Putative uncharacterized protein OS=Mus	5	3
1406	Q3USZ5	Putative uncharacterized protein (Fragme	5	3
1485	Q4VAA2	Protein CDV3 OS=Mus musculus GN=C	5	3
1635	Q60692	Proteasome subunit beta type-6 OS=Mus	5	3
1690	Q62000	Mimecan OS=Mus musculus GN=Ogn P	5	3
2011	Q8C166	Copine-1 OS=Mus musculus GN=Cpne1	5	3
2034	Q8C2S9	Putative uncharacterized protein OS=Mus	5	3
2084	Q8CG48	Structural maintenance of chromosomes p	5	3
2115	Q8K2P7	Sodium-coupled neutral amino acid trans	5	3
2263	Q99MI6	GTPase IMAP family member 3 OS=Mus	5	3
2266	Q99NB9	Splicing factor 3B subunit 1 OS=Mus mu	5	3
2277	Q9CQ65	S-methyl-5'-thioadenosine phosphorylase	5	3
2296	Q9CR26	Vacuolar protein sorting-associated protei	5	3
2337	Q9CZ69	CKLF-like MARVEL transmembrane dor	5	3
2353	Q9D0B6	Protein PBDC1 OS=Mus musculus GN=F	5	3
2390	Q9DAS9	Guanine nucleotide-binding protein G(I)/(	5	3

2420	Q9EQK5	Major vault protein OS=Mus musculus Gl	5	3
2551	Q9Z1N5	Spliceosome RNA helicase Ddx39b OS=M	5	3
398	O35235	Tumor necrosis factor ligand superfamily	5	2
489	P0C0A3	Charged multivesicular body protein 6 OS	5	2
867	Q18PJ2	Ly108 OS=Mus musculus GN=Slamf6 PE	5	2
1228	Q3U9A8	SH3 domain-binding glutamic acid-rich-li	5	2
1326	Q3UIG0	Eukaryotic translation initiation factor 3 su	5	2
1415	Q3UV17	Keratin, type II cytoskeletal 2 oral OS=Mu	5	2
1514	Q543N3	LIM and SH3 domain protein 1 OS=Mus	5	2
2136	Q8R1G6	PDZ and LIM domain protein 2 OS=Mus	5	2
2289	Q9CQT1	Methylthioribose-1-phosphate isomerase (	5	2
2314	Q9CX34	Protein SGT1 homolog OS=Mus musculu	5	2
2318	Q9CXP8	Guanine nucleotide-binding protein G(I)/(	5	2
2437	Q9ET39	SLAM family member 6 OS=Mus muscul	5	2
2452	Q9JJU8	SH3 domain-binding glutamic acid-rich-li	5	2
2524	Q9WUP7	Ubiquitin carboxyl-terminal hydrolase iso	5	2
1115	Q3TXE5	Putative uncharacterized protein OS=Mus	5	0
1372	Q3ULT8	Putative uncharacterized protein OS=Mus	5	0
1414	Q3UV15	Putative uncharacterized protein OS=Mus	5	0
1479	Q4U108	NF-kB2 splice variant 1 OS=Mus muscul	5	0
1606	Q5SUC3	Calnexin OS=Mus musculus GN=Canx Pl	5	0
2253	Q99LE6	ATP-binding cassette sub-family F memb	5	0
2516	Q9WTK5	Nuclear factor NF-kappa-B p100 subunit (	5	0
2550	Q9Z1F9	SUMO-activating enzyme subunit 2 OS=M	5	0
4	A0N8K9	T3 delta-chain (Fragment) OS=Mus musc	5	0
684	P54728	UV excision repair protein RAD23 homol	5	0
736	P62484	Abl interactor 2 OS=Mus musculus GN=A	5	0
908	Q3TCJ8	Coiled-coil domain-containing protein 69	5	0
1131	Q3U041	Putative uncharacterized protein OS=Mus	5	0
1137	Q3U0K0	Putative uncharacterized protein OS=Mus	5	0
1165	Q3U4T1	CD3 antigen delta polypeptide OS=Mus n	5	0
1174	Q3U572	Putative uncharacterized protein OS=Mus	5	0
1398	Q3UQN3	Putative uncharacterized protein OS=Mus	5	0
1671	Q61635	GTP-binding protein OS=Mus musculus (	5	0
1707	Q64151	Semaphorin-4C OS=Mus musculus GN=S	5	0
1735	Q6AXD2	Abi2 protein OS=Mus musculus GN=Abi	5	0
1737	Q6AXH6	Abi2 protein OS=Mus musculus GN=Abi	5	0
1767	Q6P5P1	CD3 antigen, delta polypeptide OS=Mus r	5	0
563	P23298	Protein kinase C eta type OS=Mus muscu	4	8
1356	Q3UKY1	Protein kinase C eta type OS=Mus muscu	4	8

1649	Q60972	Histone-binding protein RBBP4 OS=Mus	4	7
2166	Q8VIJ6	Splicing factor, proline- and glutamine-ric	4	7
2573	Z4YLB7	Neuroplastin OS=Mus musculus GN=Npt	4	7
399	O35295	Transcriptional activator protein Pur-beta	4	6
411	O35685	Nuclear migration protein nudC OS=Mus	4	6
821	P97855	Ras GTPase-activating protein-binding pr	4	6
946	Q3TGF2	Protein FAM107B OS=Mus musculus GN	4	6
973	Q3THW7	Eukaryotic translation initiation factor 3 su	4	6
988	Q3TII2	Eukaryotic translation initiation factor 3 su	4	6
1480	Q4V9X9	Rpl23a protein (Fragment) OS=Mus musc	4	6
1540	Q571F9	MKIAA4115 protein (Fragment) OS=Mus	4	6
1578	Q5M9L0	Eukaryotic translation initiation factor 3 su	4	6
1583	Q5M9M5	60S ribosomal protein L23a OS=Mus mus	4	6
1876	Q80XI4	Phosphatidylinositol 5-phosphate 4-kinase	4	6
1976	Q8BTX5	Eukaryotic translation initiation factor 3 su	4	6
2268	Q99PV0	Pre-mRNA-processing-splicing factor 8 C	4	6
2328	Q9CY61	40S ribosomal protein S24 OS=Mus musc	4	6
503	P11688	Integrin alpha-5 OS=Mus musculus GN=I	4	5
649	P46062	Signal-induced proliferation-associated pr	4	5
785	P70290	55 kDa erythrocyte membrane protein OS	4	5
1421	Q3UW40	Putative uncharacterized protein OS=Mus	4	5
1722	Q684Q6	Membrane protein, palmitoylated (Fragme	4	5
1881	Q80YP5	Integrin alpha 5 (Fibronectin receptor alph	4	5
1947	Q8BP67	60S ribosomal protein L24 OS=Mus musc	4	5
2501	Q9R0Q6	Actin-related protein 2/3 complex subunit	4	5
59	A2RSB1	Nucleosome assembly protein 1-like 4 OS	4	4
140	B7ZNL2	Nap1l4 protein OS=Mus musculus GN=N	4	4
164	D3YTQ9	40S ribosomal protein S15 OS=Mus musc	4	4
324	G3UYV7	40S ribosomal protein S28 (Fragment) OS	4	4
708	P61089	Ubiquitin-conjugating enzyme E2 N OS=I	4	4
748	P62858	40S ribosomal protein S28 OS=Mus musc	4	4
807	P97310	DNA replication licensing factor MCM2 (	4	4
826	Q00612	Glucose-6-phosphate 1-dehydrogenase X	4	4
1199	Q3U740	Eukaryotic translation initiation factor 6 C	4	4
1207	Q3U818	Eukaryotic translation initiation factor 6 C	4	4
1223	Q3U8X1	Eukaryotic translation initiation factor 3 su	4	4
1240	Q3UA13	Eukaryotic translation initiation factor 6 C	4	4
1268	Q3UBJ6	Eukaryotic translation initiation factor 6 C	4	4
1336	Q3UJN1	DNA helicase OS=Mus musculus GN=Mc	4	4
1393	Q3UPI8	Transporter OS=Mus musculus GN=Slc6a	4	4

1524	Q545K4	Eukaryotic translation initiation factor 6 C	4	4
1608	Q5SUR0	Phosphoribosylformylglycinamidine synth	4	4
1743	Q6IRT4	Eukaryotic translation initiation factor 3 su	4	4
1905	Q8BGK6	Y+L amino acid transporter 2 OS=Mus m	4	4
1908	Q8BH35	Complement component C8 beta chain OS	4	4
1975	Q8BTW2	Eukaryotic translation initiation factor 3 su	4	4
2016	Q8C1W9	Putative uncharacterized protein OS=Mus	4	4
2127	Q8QZY6	Tetraspanin-14 OS=Mus musculus GN=T	4	4
2364	Q9D1P4	Cysteine and histidine-rich domain-contai	4	4
2412	Q9DCH4	Eukaryotic translation initiation factor 3 su	4	4
2519	Q9WU28	Prefoldin subunit 5 OS=Mus musculus GN	4	4
2530	Q9WVA3	Mitotic checkpoint protein BUB3 OS=Mu	4	4
256	E9Q9H3	DnaJ homolog subfamily C member 2 OS	4	3
268	E9QLW5	Insulin-like growth factor II OS=Mus mus	4	3
315	F8WJ41	40S ribosomal protein S15a (Fragment) O	4	3
569	P24668	Cation-dependent mannose-6-phosphate re	4	3
603	P31786	Acyl-CoA-binding protein OS=Mus musc	4	3
682	P54103	DnaJ homolog subfamily C member 2 OS	4	3
698	P59325	Eukaryotic translation initiation factor 5 C	4	3
710	P61164	Alpha-centractin OS=Mus musculus GN=	4	3
749	P62869	Transcription elongation factor B polypep	4	3
841	Q05CH7	Eif5 protein OS=Mus musculus GN=Eif5	4	3
883	Q3T9G9	Putative uncharacterized protein OS=Mus	4	3
905	Q3TC45	Protein S100-A10 OS=Mus musculus GN	4	3
957	Q3THC1	Proteasome (Prosome, macropain) 26S su	4	3
1026	Q3TKY2	Putative uncharacterized protein (Fragmen	4	3
1064	Q3TPZ2	Serine/threonine-protein kinase PLK OS=	4	3
1091	Q3TV20	Putative uncharacterized protein OS=Mus	4	3
1113	Q3TXC4	Putative uncharacterized protein OS=Mus	4	3
1186	Q3U670	Putative uncharacterized protein OS=Mus	4	3
1221	Q3U8U1	Putative uncharacterized protein OS=Mus	4	3
1231	Q3U9E2	Proto-oncogene vav OS=Mus musculus G	4	3
1252	Q3UAG9	Putative uncharacterized protein OS=Mus	4	3
1253	Q3UAH3	Putative uncharacterized protein OS=Mus	4	3
1306	Q3UF03	Putative uncharacterized protein OS=Mus	4	3
1314	Q3UG81	Serine/threonine-protein kinase PLK OS=	4	3
1366	Q3ULI3	Putative uncharacterized protein OS=Mus	4	3
1377	Q3UM45	Protein phosphatase 1 regulatory subunit 7	4	3
1434	Q3UYK2	Presenilin OS=Mus musculus GN=Psen1	4	3
1453	Q3V441	Tyrosine-protein phosphatase non-recepto	4	3

1553	Q5BLK2	40S ribosomal protein S20 OS=Mus musc	4	3
1579	Q5M9L1	60S ribosomal protein L36 OS=Mus musc	4	3
1582	Q5M9M4	40S ribosomal protein S15a OS=Mus mus	4	3
1652	Q61024	Asparagine synthetase [glutamine-hydroly	4	3
1657	Q61152	Tyrosine-protein phosphatase non-recepto	4	3
1810	Q6ZWZ4	60S ribosomal protein L36 OS=Mus musc	4	3
1822	Q792Z1	MCG140784 OS=Mus musculus GN=Try	4	3
1942	Q8BMJ3	Eukaryotic translation initiation factor 1A	4	3
2113	Q8K2H4	Arf-GAP with coiled-coil, ANK repeat an	4	3
2294	Q9CR00	26S proteasome non-ATPase regulatory su	4	3
2321	Q9CXW4	60S ribosomal protein L11 OS=Mus musc	4	3
2346	Q9CZY3	Ubiquitin-conjugating enzyme E2 variant	4	3
2347	Q9CZZ4	Putative uncharacterized protein OS=Mus	4	3
2358	Q9D0R2	Threonine--tRNA ligase, cytoplasmic OS=	4	3
2454	Q9JKB1	Ubiquitin carboxyl-terminal hydrolase iso	4	3
2508	Q9R1P3	Proteasome subunit beta type-2 OS=Mus m	4	3
2511	Q9R1T2	SUMO-activating enzyme subunit 1 OS=M	4	3
2537	Q9Z0G0	PDZ domain-containing protein GIPC1 O	4	3
69	A6H6M8	Coiled-coil domain containing 50 OS=Mu	4	2
72	A7VL18	Gamma-aminobutyraldehyde dehydrogena	4	2
291	F6UK66	Coiled-coil domain-containing protein 50	4	2
515	P14094	Sodium/potassium-transporting ATPase su	4	2
606	P32067	Lupus La protein homolog OS=Mus musc	4	2
692	P56480	ATP synthase subunit beta, mitochondrial	4	2
705	P60904	DnaJ homolog subfamily C member 5 OS	4	2
740	P62814	V-type proton ATPase subunit B, brain iso	4	2
876	Q32P00	Chromobox homolog 3 (Drosophila HP1 g	4	2
944	Q3TG93	Putative uncharacterized protein OS=Mus	4	2
960	Q3THE7	Putative uncharacterized protein OS=Mus	4	2
961	Q3THH1	Putative uncharacterized protein OS=Mus	4	2
1007	Q3TJB4	Putative uncharacterized protein OS=Mus	4	2
1014	Q3TJL8	Putative uncharacterized protein OS=Mus	4	2
1019	Q3TK27	Putative uncharacterized protein OS=Mus	4	2
1049	Q3TML0	Protein disulfide-isomerase A6 OS=Mus m	4	2
1051	Q3TMT4	Putative uncharacterized protein OS=Mus	4	2
1092	Q3TV47	Sodium/potassium-transporting ATPase su	4	2
1146	Q3U1S6	Cold shock domain-containing protein E1	4	2
1287	Q3UDG0	Putative uncharacterized protein OS=Mus	4	2
1312	Q3UFZ6	Putative uncharacterized protein (Fragmen	4	2
1381	Q3UMT1	Protein phosphatase 1 regulatory subunit 1	4	2

1385	Q3UNK5	Transforming growth factor beta-1 OS=M	4	2
1542	Q571J7	Serine/threonine-protein phosphatase 2A :	4	2
1645	Q60865	Caprin-1 OS=Mus musculus GN=Caprin1	4	2
1836	Q7TNC4	Putative RNA-binding protein Luc7-like 2	4	2
1844	Q7TQI3	Ubiquitin thioesterase OTUB1 OS=Mus n	4	2
1911	Q8BHC2	2400003C14Rik protein OS=Mus muscul	4	2
1973	Q8BTU4	Putative uncharacterized protein OS=Mus	4	2
2094	Q8CI11	Guanine nucleotide-binding protein-like 3	4	2
2224	Q922R8	Protein disulfide-isomerase A6 OS=Mus r	4	2
2313	Q9CX00	IST1 homolog OS=Mus musculus GN=Ist	4	2
2320	Q9CXW3	Calcyclin-binding protein OS=Mus muscu	4	2
2389	Q9DAB4	Putative uncharacterized protein OS=Mus	4	2
2408	Q9DCC5	Cbx3 protein OS=Mus musculus GN=Cb	4	2
2465	Q9JLJ2	4-trimethylaminobutyraldehyde dehydrog	4	2
2507	Q9R1P1	Proteasome subunit beta type-3 OS=Mus r	4	2
27	A2A9X5	5'(3')-deoxyribonucleotidase, cytosolic typ	4	0
47	A2AQL0	STE20/SPS1-related proline-alanine-rich	4	0
423	O55029	Coatomer subunit beta' OS=Mus musculu	4	0
427	O55142	60S ribosomal protein L35a OS=Mus mus	4	0
451	O89110	Caspase-8 OS=Mus musculus GN=Casp8	4	0
488	P09793	Cytotoxic T-lymphocyte protein 4 OS=M	4	0
615	P35330	Intercellular adhesion molecule 2 OS=Mu	4	0
686	P54822	Adenylosuccinate lyase OS=Mus musculu	4	0
1045	Q3TMB8	Adenylosuccinate lyase OS=Mus musculu	4	0
1473	Q4FK85	Casp8 protein OS=Mus musculus GN=Ca	4	0
1741	Q6GTR6	Cytotoxic T-lymphocyte protein 4 OS=M	4	0
1832	Q7TMX1	Cytotoxic T-lymphocyte-associated protei	4	0
1961	Q8BQV7	Putative uncharacterized protein OS=Mus	4	0
2116	Q8K2Q7	BRO1 domain-containing protein BROX	4	0
2260	Q99M51	Cytoplasmic protein NCK1 OS=Mus mus	4	0
114	B2RU38	CD84 antigen OS=Mus musculus GN=Cd	4	0
137	B7ZMQ1	Cd84 protein OS=Mus musculus GN=Cd	4	0
192	D3Z7W5	CD27 antigen OS=Mus musculus GN=Cd	4	0
253	E9Q9E8	SLAM family member 5 OS=Mus muscul	4	0
302	F7D1I5	Glycophorin-C OS=Mus musculus GN=G	4	0
368	J3QNW0	DNA (cytosine-5)-methyltransferase OS=	4	0
512	P13609	Serglycin OS=Mus musculus GN=Srgn P	4	0
513	P13864	DNA (cytosine-5)-methyltransferase 1 OS	4	0
834	Q03265	ATP synthase subunit alpha, mitochondri	4	0
866	Q18PI6	SLAM family member 5 OS=Mus muscul	4	0

1130	Q3TZU7	Sorting nexin OS=Mus musculus GN=Sn:	4	0
1168	Q3U4X0	CD27 antigen OS=Mus musculus GN=Cd	4	0
1324	Q3UHZ3	DNA (cytosine-5)-methyltransferase OS=	4	0
1849	Q7TSJ0	DNA (cytosine-5)-methyltransferase OS=	4	0
2175	Q91VH2	Sorting nexin-9 OS=Mus musculus GN=S	4	0
737	P62717	60S ribosomal protein L18a OS=Mus mu:	3	7
2235	Q99J93	Interferon-induced transmembrane proteir	3	7
2418	Q9EPL8	Importin-7 OS=Mus musculus GN=Ipo7 I	3	7
318	F8WJK8	Hsc70-interacting protein OS=Mus muscu	3	6
1212	Q3U8D2	Putative uncharacterized protein OS=Mus	3	6
1239	Q3U9V1	Putative uncharacterized protein OS=Mus	3	6
2251	Q99L47	Hsc70-interacting protein OS=Mus muscu	3	6
2276	Q9CQ60	6-phosphogluconolactonase OS=Mus mu:	3	6
196	E0CYQ2	NudC domain-containing protein 2 OS=M	3	5
794	P70658	C-X-C chemokine receptor type 4 OS=Mu	3	5
1010	Q3TJG6	Putative uncharacterized protein OS=Mus	3	5
1807	Q6ZWX6	Eukaryotic translation initiation factor 2 su	3	5
2502	Q9R0Q7	Prostaglandin E synthase 3 OS=Mus musc	3	5
20	A2A813	Protein deglycase DJ-1 OS=Mus musculu	3	4
195	E0CY63	Claudin domain-containing protein 1 (Fra	3	4
310	F8WHM5	Golgi apparatus protein 1 (Fragment) OS=	3	4
406	O35593	26S proteasome non-ATPase regulatory su	3	4
614	P35293	Ras-related protein Rab-18 OS=Mus musc	3	4
789	P70423	Cationic amino acid transporter 3 OS=Mu	3	4
894	Q3TAH3	Putative uncharacterized protein OS=Mus	3	4
923	Q3TED1	S-adenosylmethionine synthase OS=Mus	3	4
1047	Q3TMH5	Putative uncharacterized protein OS=Mus	3	4
1281	Q3UD26	Putative uncharacterized protein OS=Mus	3	4
1315	Q3UGA8	Amidophosphoribosyltransferase OS=Mu	3	4
1668	Q61543	Golgi apparatus protein 1 OS=Mus muscu	3	4
1781	Q6PDI5	Proteasome-associated protein ECM29 ho	3	4
1803	Q6ZWU9	40S ribosomal protein S27 OS=Mus musc	3	4
1873	Q80X81	Acetyl-Coenzyme A acetyltransferase 3 O	3	4
2105	Q8K124	Pleckstrin homology domain-containing fa	3	4
2138	Q8R2Q8	Bone marrow stromal antigen 2 OS=Mus	3	4
2142	Q8R379	GTPase, IMAP family member 7 OS=Mu	3	4
2145	Q8R4V3	Acetyl CoA transferase-like protein OS=M	3	4
2215	Q921W7	Putative uncharacterized protein Tes OS=	3	4
2257	Q99LX0	Protein deglycase DJ-1 OS=Mus musculu	3	4
2269	Q9CPP0	Nucleoplasmin-3 OS=Mus musculus GN=	3	4

2293	Q9CQX5	Claudin domain-containing protein 1 OS=	3	4
2450	Q9JJH0	N-acetylneuraminic acid 9-phosphate synt	3	4
2461	Q9JKX6	ADP-sugar pyrophosphatase OS=Mus mu	3	4
48	A2ATI9	Golgi reassembly stacking protein 2, isofc	3	3
718	P61957	Small ubiquitin-related modifier 2 OS=Mı	3	3
787	P70349	Histidine triad nucleotide-binding protein	3	3
797	P70698	CTP synthase 1 OS=Mus musculus GN=C	3	3
820	P97825	Hematological and neurological expressec	3	3
864	Q14AI7	COP9 (Constitutive photomorphogenic) h	3	3
869	Q1HDU4	ArhGAP9 OS=Mus musculus GN=Arhga	3	3
942	Q3TG07	Putative uncharacterized protein OS=Mus	3	3
989	Q3TIJ1	Putative uncharacterized protein OS=Mus	3	3
1029	Q3TL27	Putative uncharacterized protein OS=Mus	3	3
1031	Q3TL58	Putative uncharacterized protein OS=Mus	3	3
1097	Q3TVV7	Putative uncharacterized protein OS=Mus	3	3
1109	Q3TX06	Putative uncharacterized protein OS=Mus	3	3
1171	Q3U505	Putative uncharacterized protein (Fragmeı	3	3
1301	Q3UE92	X-prolyl aminopeptidase (Aminopeptidas	3	3
1305	Q3UER8	Fibrinogen gamma chain OS=Mus muscu	3	3
1333	Q3UJ70	Putative uncharacterized protein OS=Mus	3	3
1353	Q3UKF5	X-prolyl aminopeptidase OS=Mus muscu	3	3
1399	Q3UQY7	Putative uncharacterized protein (Fragmeı	3	3
1425	Q3UWQ9	Putative uncharacterized protein OS=Mus	3	3
1519	Q544K9	Uridine 5'-monophosphate synthase OS=N	3	3
1543	Q571J8	MKIAA4207 protein (Fragment) OS=Mu	3	3
1685	Q61833	Ribophorin OS=Mus musculus GN=Rpn2	3	3
1769	Q6P7V9	Structural maintenance of chromosomes p	3	3
1817	Q78T58	Putative uncharacterized protein OS=Mus	3	3
2100	Q8JZK9	Hydroxymethylglutaryl-CoA synthase, cy	3	3
2103	Q8K0I5	3-hydroxy-3-methylglutaryl-Coenzyme A	3	3
2125	Q8QZW8	Protein Arhgap9 OS=Mus musculus GN=	3	3
2149	Q8VCM7	Fibrinogen gamma chain OS=Mus muscu	3	3
2240	Q99JX3	Golgi reassembly-stacking protein 2 OS=I	3	3
2391	Q9DAW9	Calponin-3 OS=Mus musculus GN=Cnn3	3	3
2395	Q9DBG6	Dolichyl-diphosphooligosaccharide--prot	3	3
2562	S4R1I3	Xaa-Pro aminopeptidase 1 OS=Mus musc	3	3
58	A2RS23	Peptidase D OS=Mus musculus GN=Pepc	3	2
60	A2RSY7	Perforin 1 (Pore forming protein) OS=Mu	3	2
85	B1AT92	Growth factor receptor-bound protein 2 O	3	2
144	B7ZWB3	Skap1 protein OS=Mus musculus GN=Sk	3	2

187	D3Z6Q9	Bridging integrator 2 OS=Mus musculus (	3	2
287	F6QYF8	Puromycin-sensitive aminopeptidase (Fra;	3	2
360	H3BKN0	tRNA (cytosine(34)-C(5))-methyltransfera	3	2
421	O54962	Barrier-to-autointegration factor OS=Mus	3	2
436	O70404	Vesicle-associated membrane protein 8 O:	3	2
477	P08003	Protein disulfide-isomerase A4 OS=Mus r	3	2
613	P35285	Ras-related protein Rab-22A OS=Mus mu	3	2
810	P97355	Spermine synthase OS=Mus musculus GN	3	2
851	Q08481	Platelet endothelial cell adhesion molecul	3	2
870	Q1HFZ0	tRNA (cytosine(34)-C(5))-methyltransfera	3	2
1145	Q3U1Q4	Putative uncharacterized protein OS=Mus	3	2
1158	Q3U3F4	Putative uncharacterized protein OS=Mus	3	2
1382	Q3UN35	Beta-1,4 N-acetylgalactosaminyltransfera:	3	2
1411	Q3UUV5	Src kinase-associated phosphoprotein 1 O	3	2
1629	Q60631	Growth factor receptor-bound protein 2 O	3	2
1634	Q60668-3	Isoform 3 of Heterogeneous nuclear ribon	3	2
1700	Q62422	Osteoclast-stimulating factor 1 OS=Mus r	3	2
1757	Q6P2B2	Casein kinase I isoform gamma-1 OS=Mu	3	2
1761	Q6P5B0	RRP12-like protein OS=Mus musculus G	3	2
1901	Q8BGB2	Tetratricopeptide repeat protein 7A OS=M	3	2
1924	Q8BJW6-2	Isoform 2 of Eukaryotic translation initiati	3	2
1957	Q8BQ03	DNA helicase OS=Mus musculus GN=M	3	2
1999	Q8BY71	Histone acetyltransferase type B catalytic	3	2
2017	Q8C1Z0	Putative uncharacterized protein OS=Mus	3	2
2057	Q8C9V1	Carabin OS=Mus musculus GN=Tbc1d10	3	2
2208	Q91ZX7	Prolow-density lipoprotein receptor-relate	3	2
2231	Q93092	Transaldolase OS=Mus musculus GN=Ta	3	2
2288	Q9CQR6	Serine/threonine-protein phosphatase 6 ca	3	2
2352	Q9D094	Putative uncharacterized protein OS=Mus	3	2
2384	Q9D892	Inosine triphosphate pyrophosphatase OS=	3	2
2396	Q9DBH5	Vesicular integral-membrane protein VIP:	3	2
35	A2AH85	116 kDa U5 small nuclear ribonucleoprot	3	0
52	A2AUR7	Ras suppressor protein 1 OS=Mus muscul	3	0
124	B2RXV4	Feline leukemia virus subgroup C recepto	3	0
211	E9PWZ6	Unconventional myosin-IXb OS=Mus mu	3	0
216	E9PYI8	Ubiquitin carboxyl-terminal hydrolase 14	3	0
266	E9QKV6	Unconventional myosin-IXb OS=Mus mu	3	0
307	F8WGR0	Alpha-adducin OS=Mus musculus GN=A	3	0
311	F8WHZ9	Alpha-adducin OS=Mus musculus GN=A	3	0
353	H3BJ45	Rho guanine nucleotide exchange factor 2	3	0

402	O35381	Acidic leucine-rich nuclear phosphoprotei	3	0
497	P11031	Activated RNA polymerase II transcriptio	3	0
593	P28658	Ataxin-10 OS=Mus musculus GN=Atxn1(	3	0
616	P35456-2	Isoform 2 of Urokinase plasminogen activ	3	0
668	P49442	Inositol polyphosphate 1-phosphatase OS=	3	0
830	Q01730	Ras suppressor protein 1 OS=Mus muscul	3	0
853	Q08943	FACT complex subunit SSRP1 OS=Mus r	3	0
854	Q08943-2	Isoform 2 of FACT complex subunit SSR	3	0
907	Q3TCF4	Putative uncharacterized protein OS=Mus	3	0
955	Q3THA0	Eukaryotic translation initiation factor 3 s	3	0
1024	Q3TKP3	Putative uncharacterized protein OS=Mus	3	0
1122	Q3TY45	Putative uncharacterized protein OS=Mus	3	0
1355	Q3UKS3	Single immunoglobulin and toll-interleuki	3	0
1439	Q3V005	Putative uncharacterized protein OS=Mus	3	0
1621	Q5U4D8	Sodium-dependent multivitamin transport	3	0
1623	Q5XJY5	Coatomer subunit delta OS=Mus musculu	3	0
1846	Q7TS64	Adrenergic receptor kinase, beta 1 OS=M	3	0
1872	Q80X50-2	Isoform 2 of Ubiquitin-associated protein	3	0
1891	Q811M6	Interferon gamma induced GTPase OS=M	3	0
1902	Q8BGB5	LIM domain-containing protein 2 OS=Mu	3	0
1928	Q8BK67	Protein RCC2 OS=Mus musculus GN=Rc	3	0
2111	Q8K232	Adducin 1 (Alpha) OS=Mus musculus GN	3	0
2264	Q99MK8	Beta-adrenergic receptor kinase 1 OS=Mu	3	0
2286	Q9CQM9	Glutaredoxin-3 OS=Mus musculus GN=G	3	0
2298	Q9CRB2	H/ACA ribonucleoprotein complex subun	3	0
2349	Q9D031	Ras suppressor protein 1 OS=Mus muscul	3	0
2393	Q9DB34	Charged multivesicular body protein 2a O	3	0
2411	Q9DCE9	Protein Igtp OS=Mus musculus GN=Igtp	3	0
2483	Q9QYC0	Alpha-adducin OS=Mus musculus GN=A	3	0
2489	Q9QZF2	Glypican-1 OS=Mus musculus GN=Gpc1	3	0
67	A5D6Q8	Clasp1 protein OS=Mus musculus GN=Cl	3	0
78	B1AQR8	Galectin OS=Mus musculus GN=Lgals9 I	3	0
91	B1AVH4	Coronin OS=Mus musculus GN=Coro2a I	3	0
92	B1AVH5	Coronin OS=Mus musculus GN=Coro2a I	3	0
157	B9EKT6	IQ motif containing GTPase activating pr	3	0
224	E9Q197	Glyoxalase domain-containing protein 4 C	3	0
226	E9Q2T4	Protein 4930523C07Rik OS=Mus muscul	3	0
300	F7AVU1	Cerebral cavernous malformations protein	3	0
336	G3X9T7	Galectin OS=Mus musculus GN=Lgals9 I	3	0
381	O08573	Galectin-9 OS=Mus musculus GN=Lgals9	3	0

498	P11087	Collagen alpha-1(I) chain OS=Mus muscu	3	0
564	P23492	Purine nucleoside phosphorylase OS=Mus	3	0
721	P62046	Leucine-rich repeat and calponin homolog	3	0
889	Q3TA00	Putative uncharacterized protein OS=Mus	3	0
1041	Q3TM53	Putative uncharacterized protein (Fragmer	3	0
1159	Q3U3P5	Putative uncharacterized protein OS=Mus	3	0
1388	Q3UP01	Putative uncharacterized protein (Fragmer	3	0
1510	Q543E3	Suppressor of tumorigenicity 14 protein h	3	0
1513	Q543K9	Purine nucleoside phosphorylase OS=Mus	3	0
1516	Q543Y7	Protein kinase C and casein kinase substra	3	0
1556	Q5D0E4	SH2 domain protein 2A OS=Mus musculu	3	0
1738	Q6DFW4	Nucleolar protein 58 OS=Mus musculus C	3	0
1770	Q6P8X1	Sorting nexin-6 OS=Mus musculus GN=S	3	0
1893	Q8BFQ3	Ovarian cancer G-protein coupled recepto	3	0
2041	Q8C4J7	Transducin beta-like protein 3 OS=Mus m	3	0
2112	Q8K242	Tgtp protein OS=Mus musculus PE=2 SV	3	0
2118	Q8K2Y9	Cerebral cavernous malformations protein	3	0
2272	Q9CPV4	Glyoxalase domain-containing protein 4 C	3	0
2478	Q9QXK9	SH2 domain-containing protein 2A OS=M	3	0
201	E9PV24	Fibrinogen alpha chain OS=Mus musculus	2	7
1864	Q80UL9	Junctional adhesion molecule-like OS=Mu	2	6
2443	Q9JI48	Placenta-specific gene 8 protein OS=Mus	2	6
320	G3UXL2	Protein Prps1l3 OS=Mus musculus GN=P	2	5
469	P05533	Lymphocyte antigen 6A-2/6E-1 OS=Mus	2	5
1117	Q3TXL0	Putative uncharacterized protein (Fragmer	2	5
1302	Q3UEG9	Putative uncharacterized protein OS=Mus	2	5
1467	Q4FJZ3	Ero1l protein OS=Mus musculus GN=Ero	2	5
1472	Q4FK57	ERO1-like protein alpha OS=Mus muscul	2	5
1604	Q5SS83	Flotillin 2, isoform CRA_a OS=Mus musc	2	5
1630	Q60634	Flotillin-2 OS=Mus musculus GN=Flot2 I	2	5
2014	Q8C1L7	40S ribosomal protein S21 OS=Mus musc	2	5
2287	Q9CQR2	40S ribosomal protein S21 OS=Mus musc	2	5
2402	Q9DC36	Putative uncharacterized protein OS=Mus	2	5
64	A3KGQ6	Actin-related protein 2/3 complex subunit	2	4
145	B7ZWC0	N-myc downstream regulated gene 1 OS=	2	4
400	O35344	Importin subunit alpha-4 OS=Mus muscul	2	4
441	O88543	COP9 signalosome complex subunit 3 OS	2	4
912	Q3TD08	Putative uncharacterized protein OS=Mus	2	4
914	Q3TD51	Putative uncharacterized protein OS=Mus	2	4
1526	Q545R3	N-myc downstream regulated gene 1 OS=	2	4

1537	Q570Z8	MKIAA4114 protein (Fragment) OS=Mu:	2	4
1823	Q7M6Y3	Phosphatidylinositol-binding clathrin asse	2	4
2213	Q921G6	Leucine-rich repeat and calponin homolog	2	4
2261	Q99M54	Cell division cycle-associated protein 3 O	2	4
2344	Q9CZV9	Putative uncharacterized protein OS=Mus	2	4
2379	Q9D771	Transmembrane protein 206 OS=Mus mu:	2	4
2414	Q9DCT8	Cysteine-rich protein 2 OS=Mus musculu:	2	4
2432	Q9EST5-2	Isoform 2 of Acidic leucine-rich nuclear p	2	4
279	E9QPI5	Sister chromatid cohesion protein PDS5 h	2	3
652	P46664	Adenylosuccinate synthetase isozyme 2 O	2	3
707	P61082	NEDD8-conjugating enzyme Ubc12 OS=]	2	3
761	P63024	Vesicle-associated membrane protein 3 O:	2	3
836	Q04750	DNA topoisomerase 1 OS=Mus musculus	2	3
887	Q3T9U1	MOB kinase activator 1B OS=Mus muscu	2	3
1215	Q3U8L9	Putative uncharacterized protein OS=Mus	2	3
1293	Q3UDM0	MOB kinase activator 1B OS=Mus muscu	2	3
1545	Q58E29	Proteolipid protein 2 OS=Mus musculus C	2	3
1694	Q62318	Transcription intermediary factor 1-beta C	2	3
1845	Q7TQI7	Ankyrin repeat and BTB/POZ domain-con	2	3
1913	Q8BHL3	TBC1 domain family member 10B OS=M	2	3
2093	Q8CHP8	Phosphoglycolate phosphatase OS=Mus n	2	3
2181	Q91VW3	SH3 domain-binding glutamic acid-rich-li	2	3
2216	Q921Y0	MOB kinase activator 1A OS=Mus muscu	2	3
2247	Q99KP6	Pre-mRNA-processing factor 19 OS=Mus	2	3
2442	Q9JHU9	Inositol-3-phosphate synthase 1 OS=Mus	2	3
2444	Q9JIF7	Coatomer subunit beta OS=Mus musculus	2	3
2477	Q9QXB9	Developmentally-regulated GTP-binding]	2	3
2510	Q9R1Q7	Proteolipid protein 2 OS=Mus musculus C	2	3
109	B2RQA7	Ncapg protein OS=Mus musculus GN=Nc	2	2
198	E9PUD2	Dynamin-1-like protein OS=Mus musculu	2	2
208	E9PWG6	Protein Ncapg OS=Mus musculus GN=Nc	2	2
303	F8VPU2	FERM, RhoGEF and pleckstrin domain-c	2	2
331	G3X8Y3	N-alpha-acetyltransferase 15, NatA auxili:	2	2
359	H3BKH6	S-formylglutathione hydrolase OS=Mus n	2	2
623	P38647	Stress-70 protein, mitochondrial OS=Mus	2	2
627	P39429	TNF receptor-associated factor 2 OS=Mu:	2	2
628	P39429-2	Isoform 2 of TNF receptor-associated fact	2	2
650	P46460	Vesicle-fusing ATPase OS=Mus musculu	2	2
683	P54116	Erythrocyte band 7 integral membrane prc	2	2
796	P70677	Caspase-3 OS=Mus musculus GN=Casp3	2	2

822	P98064	Mannan-binding lectin serine protease 1 C	2	2
1054	Q3TN07	Vacuolar protein sorting 4b (Yeast) OS=M	2	2
1065	Q3TPZ5	Dynactin 2 OS=Mus musculus GN=Dctn2	2	2
1107	Q3TWR1	Putative uncharacterized protein OS=Mus	2	2
1123	Q3TYJ0	Putative uncharacterized protein OS=Mus	2	2
1308	Q3UF95	Large proline-rich protein BAG6 OS=Mu	2	2
1344	Q3UJW9	Putative uncharacterized protein OS=Mus	2	2
1444	Q3V1M8	Putative uncharacterized protein OS=Mus	2	2
1515	Q543N7	Protein kinase C and casein kinase II subs	2	2
1539	Q571D6	MKIAA0281 protein (Fragment) OS=Mu	2	2
1712	Q64433	10 kDa heat shock protein, mitochondrial	2	2
1717	Q64727	Vinculin OS=Mus musculus GN=Vcl PE=	2	2
1827	Q7TML3	Solute carrier family 35 member F2 OS=M	2	2
1865	Q80UM3	N-alpha-acetyltransferase 15, NatA auxili	2	2
1956	Q8BPU7	Engulfment and cell motility protein 1 OS	2	2
2050	Q8C6X9	Putative uncharacterized protein OS=Mus	2	2
2153	Q8VDJ3	Vigilin OS=Mus musculus GN=Hdlbp PE	2	2
2366	Q9D1R9	60S ribosomal protein L34 OS=Mus musc	2	2
2427	Q9ES52	Phosphatidylinositol 3,4,5-trisphosphate 5	2	2
2430	Q9ES94	Cathepsin Z OS=Mus musculus GN=Ctsz	2	2
2445	Q9JII6	Alcohol dehydrogenase [NADP(+)] OS=M	2	2
2526	Q9WUU7	Cathepsin Z OS=Mus musculus GN=Ctsz	2	2
2553	Q9Z1R2	Large proline-rich protein BAG6 OS=Mu	2	2
44	A2AQ41	Formin-binding protein 1 OS=Mus muscu	2	0
45	A2AQ42	Formin-binding protein 1 OS=Mus muscu	2	0
169	D3YYK8	Microtubule-associated protein RP/EB far	2	0
214	E9PYG5	Gamma-parvin OS=Mus musculus GN=P	2	0
247	E9Q6X0	Microtubule-associated protein RP/EB far	2	0
295	F6XC25	Coiled-coil and C2 domain-containing pro	2	0
348	G5E8S8	MAGUK p55 subfamily member 7 OS=M	2	0
429	O70133	ATP-dependent RNA helicase A OS=Mus	2	0
440	O88456	Calpain small subunit 1 OS=Mus musculu	2	0
552	P19324	Serpin H1 OS=Mus musculus GN=Serpin	2	0
617	P35550	rRNA 2'-O-methyltransferase fibrillarin O	2	0
659	P47941	Crk-like protein OS=Mus musculus GN=(	2	0
772	P63254	Cysteine-rich protein 1 OS=Mus musculu	2	0
844	Q05D44	Eukaryotic translation initiation factor 5B	2	0
1008	Q3TJF2	Obg-like ATPase 1 OS=Mus musculus GI	2	0
1013	Q3TJK3	Putative uncharacterized protein OS=Mus	2	0
1050	Q3TMM5	Putative uncharacterized protein OS=Mus	2	0

1056	Q3TN44	Putative uncharacterized protein OS=Mus	2	0
1057	Q3TN94	Putative uncharacterized protein OS=Mus	2	0
1105	Q3TWG9	Putative uncharacterized protein OS=Mus	2	0
1227	Q3U955	Putative uncharacterized protein OS=Mus	2	0
1342	Q3UJS2	Putative uncharacterized protein OS=Mus	2	0
1445	Q3V1Z7	Putative uncharacterized protein OS=Mus	2	0
1488	Q501J7	Phosphatase and actin regulator 4 OS=Mu	2	0
1711	Q64374	Regucalcin OS=Mus musculus GN=Rgn I	2	0
1758	Q6P3B4	Cysteine-rich protein 1 (Intestinal) OS=M	2	0
1773	Q6P9L6	Kinesin-like protein KIF15 OS=Mus musc	2	0
1839	Q7TPN1	Cc2d1b protein (Fragment) OS=Mus musc	2	0
1983	Q8BV87	Putative uncharacterized protein OS=Mus	2	0
1984	Q8BVD5	MAGUK p55 subfamily member 7 OS=M	2	0
1988	Q8BVU9	Putative uncharacterized protein OS=Mus	2	0
2097	Q8CIG8	Protein arginine N-methyltransferase 5 OS	2	0
2120	Q8K2Z4-2	Isoform 2 of Condensin complex subunit	2	0
2178	Q91VR5	ATP-dependent RNA helicase DDX1 OS=	2	0
2292	Q9CQX2	Cytochrome b5 type B OS=Mus musculus	2	0
2335	Q9CZ30	Obg-like ATPase 1 OS=Mus musculus GN	2	0
3	A0JNY7	Eukaryotic translation initiation factor 4, g	2	0
77	B0V2N1	Receptor-type tyrosine-protein phosphatas	2	0
182	D3Z4J5	Golgi to ER traffic protein 4 homolog (Fra	2	0
220	E9PZC3	Flavin reductase (NADPH) OS=Mus musc	2	0
340	G3XA17	Eukaryotic translation initiation factor 4 g	2	0
357	H3BK65	Epidermal growth factor receptor substrat	2	0
641	P42567	Epidermal growth factor receptor substrat	2	0
910	Q3TCR9	Putative uncharacterized protein OS=Mus	2	0
1070	Q3TRH2	26S proteasome non-ATPase regulatory su	2	0
1133	Q3U0C0	Cytohesin 4 OS=Mus musculus GN=Cyth	2	0
1191	Q3U6G1	Biliverdin reductase B (Flavin reductase (	2	0
1211	Q3U8A6	Putative uncharacterized protein OS=Mus	2	0
1213	Q3U8F5	Putative uncharacterized protein OS=Mus	2	0
1701	Q62448	Eukaryotic translation initiation factor 4 g	2	0
1704	Q62523	Zyxin OS=Mus musculus GN=Zyx PE=1	2	0
1775	Q6P9P6	Kinesin-like protein KIF11 OS=Mus musc	2	0
1843	Q7TQE2	Zyx protein OS=Mus musculus GN=Zyx	2	0
1990	Q8BW03	ELAV-like protein OS=Mus musculus GN	2	0
2063	Q8CBC8	Branched-chain-amino-acid aminotransfer	2	0
2065	Q8CBM0	Putative uncharacterized protein OS=Mus	2	0
2071	Q8CD55	Putative uncharacterized protein OS=Mus	2	0

2191	Q91XH5	Sepiapterin reductase OS=Mus musculus	2	0
2363	Q9D1H7	Golgi to ER traffic protein 4 homolog OS	2	0
2387	Q9D8W5	26S proteasome non-ATPase regulatory su	2	0
2304	Q9CWF2	Tubulin beta-2B chain OS=Mus musculus	0	58
10	A1L3C9	Tumor necrosis factor receptor superfamil	0	17
450	O89103	Complement component C1q receptor OS	0	9
237	E9Q4S7	Receptor-type tyrosine-protein phosphatas	0	8
640	P42232	Signal transducer and activator of transcri	0	6
852	Q08879-2	Isoform C of Fibulin-1 OS=Mus musculus	0	6
1383	Q3UN51	Syntenin-2 OS=Mus musculus GN=Sdcbp	0	5
1886	Q810B2	Semaphorin M (Fragment) OS=Mus musc	0	5
2179	Q91VR8	Protein BRICK1 OS=Mus musculus GN=	0	5
2359	Q9D176	Sushi domain-containing protein 3 OS=M	0	5
2545	Q9Z123	Semaphorin-4F OS=Mus musculus GN=S	0	5
732	P62315	Small nuclear ribonucleoprotein Sm D1 O	0	4
801	P83940	Transcription elongation factor B polypep	0	4
2291	Q9CQW9	Interferon-induced transmembrane protein	0	4
166	D3YUT3	40S ribosomal protein S19 (Fragment) OS	0	3
184	D3Z5R8	40S ribosomal protein S19 (Fragment) OS	0	3
188	D3Z722	40S ribosomal protein S19 OS=Mus musc	0	3
189	D3Z780	Translation initiation factor eIF-2B subun	0	3
285	F6QL70	Protein Gm17669 OS=Mus musculus GN	0	3
396	O35127	Protein C10 OS=Mus musculus GN=Grcc	0	3
658	P47915	60S ribosomal protein L29 OS=Mus musc	0	3
1142	Q3U1H7	Sorting nexin OS=Mus musculus GN=Snx	0	3
1392	Q3UPB9	Sorting nexin OS=Mus musculus GN=Snx	0	3
1586	Q5M9P3	Rps19 protein (Fragment) OS=Mus muscu	0	3
1656	Q61098	Interleukin-18 receptor 1 OS=Mus muscul	0	3
1658	Q61166	Microtubule-associated protein RP/EB fan	0	3
1666	Q61398	Procollagen C-endopeptidase enhancer 1	0	3
1680	Q61749	Translation initiation factor eIF-2B subun	0	3
1708	Q64281	Leukocyte immunoglobulin-like receptor	0	3
1852	Q7TT37	Elongator complex protein 1 OS=Mus mu	0	3
2052	Q8C788	Sorting nexin OS=Mus musculus GN=Snx	0	3
2053	Q8C845	EF-hand domain-containing protein D2 O	0	3
2143	Q8R3Q1	Tissue factor OS=Mus musculus GN=F3	0	3
2227	Q923G2	DNA-directed RNA polymerases I, II, and	0	3
2345	Q9CZX8	40S ribosomal protein S19 OS=Mus musc	0	3
2368	Q9D2R0	Acetoacetyl-CoA synthetase OS=Mus mu	0	3
2464	Q9JLF6	Protein-glutamine gamma-glutamyltransf	0	3

68	A6H667	Protein kinase C theta type OS=Mus musc	0	2
98	B1AZI6	THO complex subunit 2 OS=Mus muscul	0	2
193	D6RH37	Serine/threonine-protein kinase N1 OS=M	0	2
239	E9Q5B5	Hexokinase OS=Mus musculus GN=Hk2	0	2
258	E9QAU9	Leucine-rich repeat and calponin homolog	0	2
379	O08528	Hexokinase-2 OS=Mus musculus GN=Hk	0	2
784	P70268	Serine/threonine-protein kinase N1 OS=M	0	2
804	P84104	Serine/arginine-rich splicing factor 3 OS=	0	2
994	Q3TIR2	Putative uncharacterized protein OS=Mus	0	2
1129	Q3TZP3	Putative uncharacterized protein OS=Mus	0	2
1451	Q3V3W7	Putative uncharacterized protein OS=Mus	0	2
1662	Q61206	Platelet-activating factor acetylhydrolase I	0	2
1910	Q8BH61	Coagulation factor XIII A chain OS=Mus	0	2
1987	Q8BVU0	Leucine-rich repeat and calponin homolog	0	2
2003	Q8BYZ1	ABI gene family member 3 OS=Mus mus	0	2
2004	Q8BZP5	Putative uncharacterized protein OS=Mus	0	2
2137	Q8R1Q8	Cytoplasmic dynein 1 light intermediate c	0	2
2226	Q923F1	Chloride channel, nucleotide-sensitive, 1A	0	2
2256	Q99LN9	Deoxyhypusine hydroxylase OS=Mus mu	0	2
2258	Q99M31	Heat shock 70 kDa protein 14 OS=Mus m	0	2
2279	Q9CQC6	Basic leucine zipper and W2 domain-cont	0	2
2281	Q9CQE8	UPF0568 protein C14orf166 homolog OS	0	2
2377	Q9D6W4	Putative uncharacterized protein OS=Mus	0	2
2479	Q9QXS1	Plectin OS=Mus musculus GN=Plec PE=1	0	2
2504	Q9R190	Metastasis-associated protein MTA2 OS=	0	2
2549	Q9Z1D2	Breast cancer type 1 susceptibility protein	0	2

**Biological process (BP)**	BP 3SI	BP 2SI
cellular process (GO:0009987)	32.30%	31.80%
metabolic process (GO:0008152)	23.80%	23.50%
response to stimulus (GO:0050896)	9.90%	8.30%
cellular component organization or biogenesis (GO:0071840)	8.50%	9.70%
immune system process (GO:0002376)	5.80%	5.40%
localization (GO:0051179)	5.40%	6.10%
biological regulation (GO:0065007)	4.90%	4.30%
developmental process (GO:0032502)	4.00%	4.30%
biological adhesion (GO:0022610)	2.20%	1.80%
multicellular organismal process (GO:0032501)	1.30%	2.90%
locomotion (GO:0040011)	1.30%	1.10%
reproduction (GO:0000003)	0.40%	0.70%

**Cellular component (CC)**	CC 3SI	CC 2SI
cell part (GO:0044464)	44.90%	43.60%
organelle (GO:0043226)	26.20%	23.60%
macromolecular complex (GO:0032991)	16.80%	17.90%
membrane (GO:0016020)	7.50%	10.00%
extracellular region (GO:0005576)	3.70%	2.90%

**Protein Class (PC)**	PC 3SI	PC 2SI
nucleic acid binding (PC00171)	14.00%	14.50%
cytoskeletal protein (PC00085)	13.20%	11.60%
enzyme modulator (PC00095)	13.20%	13.30%
hydrolase (PC00121)	10.10%	11.60%
signaling molecule (PC00207)	9.30%	9.80%
receptor (PC00197)	5.40%	4.60%
transporter (PC00227)	4.70%	5.20%
transferase (PC00220)	4.70%	4.00%
oxidoreductase (PC00176)	4.70%	3.50%
ligase (PC00142)	3.90%	3.50%
cell adhesion molecule (PC00069)	3.10%	3.50%
defense/immunity protein (PC00090)	3.10%	2.30%
transcription factor (PC00218)	3.10%	3.50%
transfer/carrier protein (PC00219)	2.30%	1.20%
extracellular matrix protein (PC00102)	1.60%	0.60%

calcium-binding protein (PC00060)	1.60%	1.70%
chaperone (PC00072)	0.80%	0.60%
structural protein (PC00211)	0.80%	1.20%

Supplementary table-3 Unique Proteins Induced by IL-12

	Protein names
Q9CWF2	Tubulin beta-2B chain
A1L3C9	Tumor necrosis factor receptor superfamily, member 8
O89103	Complement component C1q receptor (C1q/MBL/SPA receptor) (C1qR(p)) (C1qRp) (Cell surface antige
E9Q4S7	Receptor-type tyrosine-protein phosphatase eta
P42232	Signal transducer and activator of transcription 5B
Q08879-2	Fibulin-1 (FIBL-1) (Basement-membrane protein 90) (BM-90)
Q3UN51	Putative uncharacterized protein
Q810B2	Semaphorin M (Fragment)
Q91VR8	Protein BRICK1 (BRK1)
Q9D176	Sushi domain-containing protein 3
Q9Z123	Semaphorin-4F (Semaphorin-W) (Sema W)
P62315	Small nuclear ribonucleoprotein Sm D1 (Sm-D1) (Sm-D autoantigen) (snRNP core protein D1)
P83940	Elongin-C (EloC) (Elongin 15 kDa subunit) (RNA polymerase II transcription factor SIII subunit C) (SII
Q9CQW9	Interferon-induced transmembrane protein 3 (Dispanin subfamily A member 2b) (DSPA2b) (Fragilis prot
D3YUT3	40S ribosomal protein S19 (Fragment)
D3Z5R8	40S ribosomal protein S19 (Fragment)
D3Z722	40S ribosomal protein S19
D3Z780	Translation initiation factor eIF-2B subunit delta
F6QL70	60S ribosomal protein L29
O35127	Protein C10
P47915	60S ribosomal protein L29
Q3U1H7	Sorting nexin
Q3UPB9	Sorting nexin
Q5M9P3	Rps19 protein (Fragment)
Q61098	Interleukin-18 receptor 1 (IL-18R-1) (IL-18R1) (CD218 antigen-like family member A) (IL1 receptor-rel
Q61166	Microtubule-associated protein RP/EB family member 1 (APC-binding protein EB1) (End-binding protei
Q61398	Procollagen C-endopeptidase enhancer 1 (P14) (Procollagen COOH-terminal proteinase enhancer 1) (PC
Q61749	Translation initiation factor eIF-2B subunit delta (eIF-2B GDP-GTP exchange factor subunit delta)
Q64281	Leukocyte immunoglobulin-like receptor subfamily B member 4 (Mast cell surface glycoprotein Gp49B)
Q7TT37	Elongator complex protein 1 (ELP1) (IkappaB kinase complex-associated protein) (IKK complex-associa
Q8C788	Sorting nexin
Q8C845	EF-hand domain-containing protein D2 (Efhd2 protein) (Putative uncharacterized protein)
Q8R3Q1	Tissue factor (TF)
Q923G2	DNA-directed RNA polymerases I, II, and III subunit RPABC3 (RNA polymerases I, II, and III subunit
Q9CZX8	40S ribosomal protein S19
Q9D2R0	Acetoacetyl-CoA synthetase (EC 6.2.1.16)
Q9JLF6	Protein-glutamine gamma-glutamyltransferase K (EC 2.3.2.13) (Epidermal TGase) (Transglutaminase K)
A6H667	Protein kinase C theta type (EC 2.7.11.13) (nPKC-theta)
B1AZI6	THO complex subunit 2 (Tho2)
D6RH37	Serine/threonine-protein kinase N1

E9Q5B5	Hexokinase-2
E9QAU9	Leucine-rich repeat and calponin homology domain-containing protein 3
O08528	Hexokinase-2 (EC 2.7.1.1) (Hexokinase type II) (HK II)
P70268	Serine/threonine-protein kinase N1 (EC 2.7.11.13) (Protein kinase C-like 1) (Protein kinase C-like PKN)
P84104	Serine/arginine-rich splicing factor 3 (Pre-mRNA-splicing factor SRP20) (Protein X16) (Splicing factor,
Q3TIR2	Putative uncharacterized protein
Q3TZP3	Putative uncharacterized protein
Q3V3W7	Putative uncharacterized protein
Q61206	Platelet-activating factor acetylhydrolase IB subunit beta (EC 3.1.1.47) (PAF acetylhydrolase 30 kDa sub
Q8BH61	Coagulation factor XIII A chain (Coagulation factor XIIIa) (EC 2.3.2.13) (Protein-glutamine gamma-glut
Q8BVU0	Leucine-rich repeat and calponin homology domain-containing protein 3
Q8BYZ1	ABI gene family member 3 (New molecule including SH3) (Nesh)
Q8BZP5	Putative uncharacterized protein
Q8R1Q8	Cytoplasmic dynein 1 light intermediate chain 1 (Dynein light chain A) (DLC-A) (Dynein light intermedi
Q923F1	Chloride channel, nucleotide-sensitive, 1A (Chloride channel, nucleotide-sensitive, 1A, isoform CRA_b)
Q99LN9	Deoxyhypusine hydroxylase (DOHH) (EC 1.14.99.29) (Deoxyhypusine dioxygenase) (Deoxyhypusine m
Q99M31	Heat shock 70 kDa protein 14 (NST-1) (hsr.1)
Q9CQC6	Basic leucine zipper and W2 domain-containing protein 1
Q9CQE8	UPF0568 protein C14orf166 homolog
Q9D6W4	Putative uncharacterized protein
Q9QXS1	Plectin (PCN) (PLTN) (Plectin-1) (Plectin-6)
Q9R190	Metastasis-associated protein MTA2 (Metastasis-associated 1-like 1)
Q9Z1D2	Breast cancer type 1 susceptibility protein homolog (EC 2.3.2.27)

Supplementary Table-4 Molecular and Cellular Functions of IL-12-induced Unique Proteins

Functions	Genes	P value
**Cellular Movement**	18	2.88E-02 – 6.26E-05
**Cell Morphology**	16	2.84E-02 – 7.14E-05
**Cell Death and Survival**	22	2.62E-02 – 4.12E-04
**Cellular Function and Maintenance**	26	2.84E-02 – 4.80E-04
**Molecular Transport**	6	2.19E-02 – 5.22E-04
